# Efficient biosynthesis of resveratrol via combining phenylalanine and tyrosine pathways in *Saccharomyces cerevisiae*

**DOI:** 10.1186/s12934-023-02055-9

**Published:** 2023-03-08

**Authors:** Lijun Meng, Mengxue Diao, Qingyan Wang, Longyun Peng, Jianxiu Li, Nengzhong Xie

**Affiliations:** State Key Laboratory of NonFood Biomass and Enzyme Technology, Guangxi Key Laboratory of Bio-Refinery, National Engineering Research Center for Non-Food Biorefinery, Guangxi Biomass Engineering Technology Research Center, Guangxi Academy of Sciences, 98 Daling Road, Nanning, 530007 China

**Keywords:** Resveratrol, *Saccharomyces cerevisiae*, Phenylalanine/tyrosine ammonia lyase, Non-auxotrophic strain, Metabolic engineering

## Abstract

**Background:**

Resveratrol is a commercially available stilbenoid widely used as dietary supplements, functional food ingredients, and cosmetic ingredients due to its diverse physiological activities. The production of resveratrol in microorganisms provides an ideal source that reduces the cost of resveratrol, but the titer in *Saccharomyces cerevisiae* was still much lower than that in other hosts.

**Results:**

To achieve enhanced production of resveratrol in *S. cerevisiae*, we constructed a biosynthetic pathway via combining phenylalanine and tyrosine pathways by introducing a bi-functional phenylalanine/tyrosine ammonia lyase from *Rhodotorula toruloides*. The combination of phenylalanine pathway with tyrosine pathway led to a 462% improvement of resveratrol production in yeast extract peptone dextrose (YPD) medium with 4% glucose, suggesting an alternative strategy for producing *p*-coumaric acid-derived compounds. Then the strains were further modified by integrating multi-copy biosynthetic pathway genes, improving metabolic flux to aromatic amino acids and malonyl-CoA, and deleting by-pathway genes, which resulted in 1155.0 mg/L resveratrol in shake flasks when cultured in YPD medium. Finally, a non-auxotrophic strain was tailored for resveratrol production in minimal medium without exogenous amino acid addition, and the highest resveratrol titer (4.1 g/L) ever reported was achieved in *S. cerevisiae* to our knowledge.

**Conclusions:**

This study demonstrates the advantage of employing a bi-functional phenylalanine/tyrosine ammonia lyase in the biosynthetic pathway of resveratrol, suggesting an effective alternative in the production of *p*-coumaric acid-derived compounds. Moreover, the enhanced production of resveratrol in *S. cerevisiae* lays a foundation for constructing cell factories for various stilbenoids.

**Graphical Abstract:**

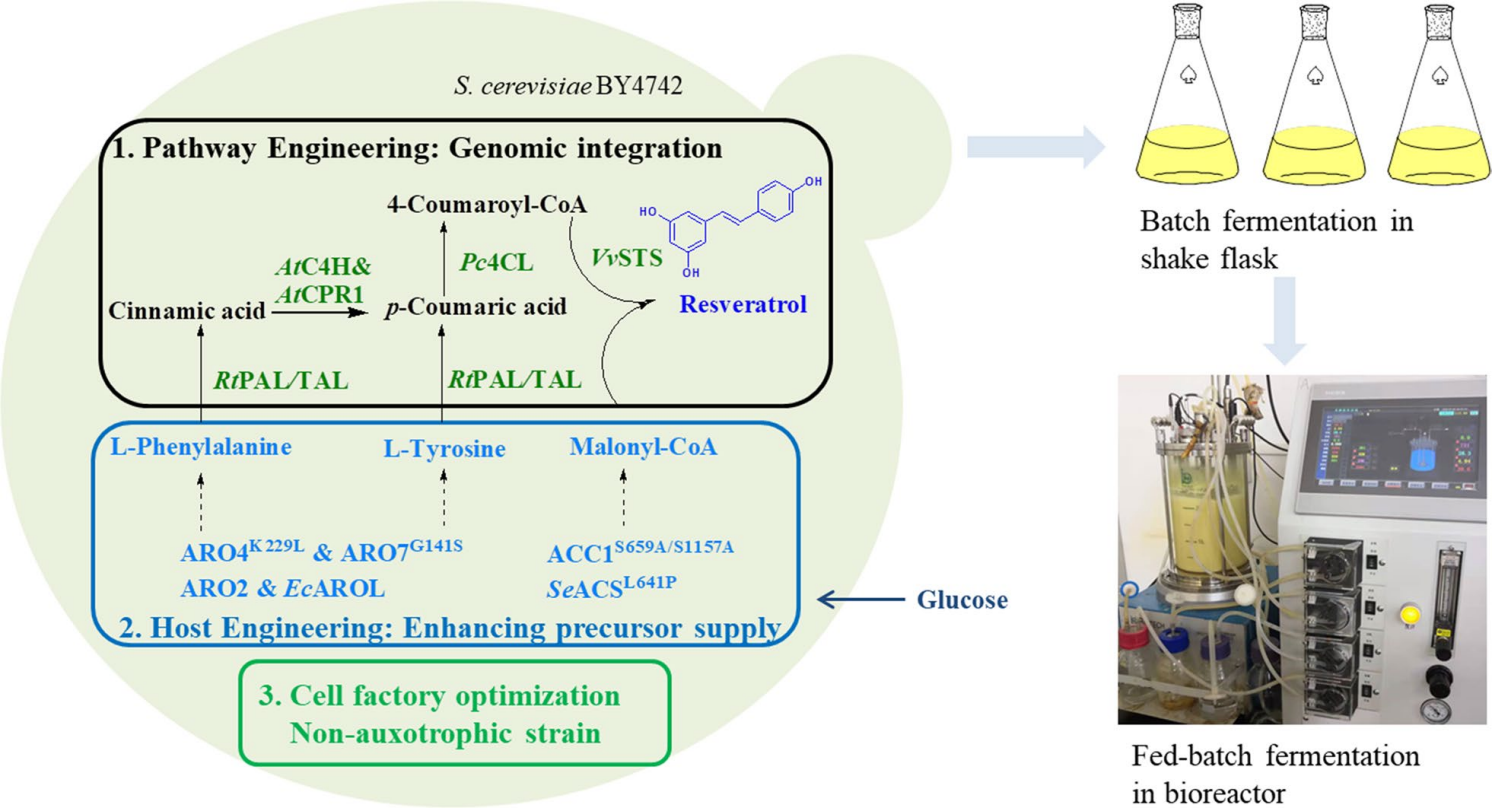

**Supplementary Information:**

The online version contains supplementary material available at 10.1186/s12934-023-02055-9.

## Background

Resveratrol is a naturally occurring plant polyphenolic compound that belongs to the stilbenoid group [1]. It has become one of the most extensively studied stilbenoids due to its remarkable biological activities, including anti-inflammatory, antimicrobial, anti-cancer, anti-aging and many other properties [2]. Moreover, resveratrol derivatives, such as pterostilbene, piceatannol, piceid, and viniferin, are bioactive compounds with diverse pharmacological activities and health benefits [3–5]. Therefore, resveratrol and its derivatives show great potential in the health, diet, and cosmetic industries [6, 7]. Considering the environmental and economic benefits, biotechnological production of resveratrol provides a promising alternative to chemical synthesis or plant extraction [8–11].

**Fig. 1:**
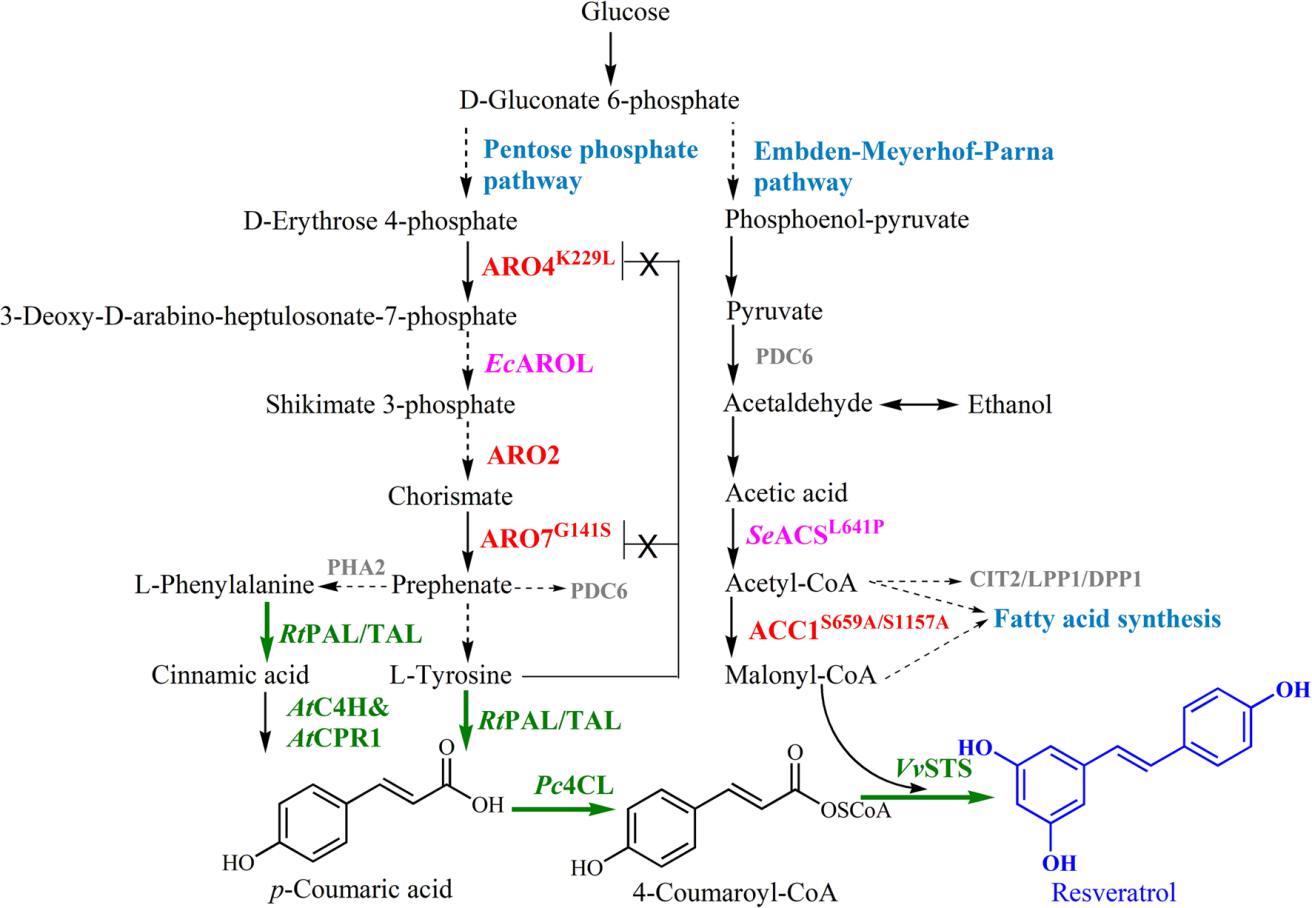
De novo biosynthetic pathway for resveratrol production in engineered *S. cerevisiae*. Both tyrosine and phenylalanine were converted by bi-functional phenylalanine/tyrosine lyase from *R. toruloides* (*Rt*PAL/TAL). ARO4^K229L^: 3-deoxy-D-arabinoheptulosonate-7-phosphate (DAHP) synthase mutant; *Ec*AROL: shikimate kinase from *E. coli*; ARO2: chorismate synthase; ARO7^G141S^: chorismate mutase mutant; *Se*ACS^L641P^: acetyl-CoA synthase mutant from *S. enterica*; ACC1^S659A/S1157A^: acetyl-CoA carboxylase mutant; *At*C4H&*At*CPR1: cinnamate-4-hydroxylase and cytochrome P450 reductase from *A. thaliana*; *Pc*4CL: *p*-coumaroyl coenzyme A ligase from *P. crispum*; *Vv*STS: stilbene synthase from *V. vinifera*. The solid arrow indicated one enzymatic step, the dash arrow indicated multiple enzymatic steps, and the green arrow in bold indicated multi-copy integration of pathway genes. The green letters represent heterogeneous pathway of resveratrol, others represent the genes involved in host engineering, with the red ones from *S. cerevisiae* were overexpressed, purple ones from other organisms were overexpressed, and grey ones from *S. cerevisiae* were knocked out

In plants, the resveratrol biosynthetic pathway begins with L-phenylalanine [12], as shown in Fig. 1. Firstly, phenylalanine ammonia lyase transforms L-phenylalanine into cinnamic acid, which is further converted into *p*-coumaric acid catalyzed by cinnamate-4-hydroxylase. Alternatively, tyrosine ammonia lyase can directly transform L-tyrosine into *p*-coumaric acid in a single step. Secondly, *p*-coumaroyl coenzyme A ligase catalyzes the conversion of *p*-coumaric acid to form *p*-coumaroyl-CoA. In the final step, resveratrol is generated by the condensation of *p*-coumaroyl-CoA with three units of malonyl-CoA by stilbene synthase. Malonyl-CoA is the other prime precursor involved in resveratrol biosynthesis, which is produced from acetyl-CoA by the catalysis of acetyl-CoA carboxylase [13].

The constructions of microbial cell factories for resveratrol production have been achieved in different microorganisms (Additional file 1: Table S1), such as *Escherichia coli*, *Saccharomyces cerevisiae* and *Yarrowia lipolytica* [9, 14]. As a well-studied model microorganism, *S. cerevisiae* has become one of the most widely used microbial host to produce various chemicals, due to its well-characterized genetic background, well-established tools available for genetic manipulation, superior stress tolerance, and excellent fermentation properties [15, 16]. Various natural products have been reported to be biosynthesized in *S. cerevisiae* for industrial and pharmaceutical applications [17–19]. Since heterologous production of resveratrol was first published in 2003 [20], metabolic engineering strategies, including pathway engineering, host engineering and enzyme engineering, have been widely employed to enhance resveratrol productions [21–23]. However, only limited investigations with regards to de novo production of resveratrol in *S. cerevisiae* were reported [24–28]. In these reports, resveratrol production via either tyrosine pathway or phenylalanine pathway can be significantly improved by multi-copy integration of the entire resveratrol biosynthetic pathway and overexpression of non-pathway genes involved in precursor supply. Other reports mainly focused on the utilization of hydrothermally pretreated *Eucalyptus* wood [26], lactose-rich wastes [27], wine wastes [28] while little effort was made in engineering the resveratrol biosynthetic pathway. So far, the highest titer achieved in *S. cerevisiae* reported in scientific publications was 0.8 g/L, which was much lower than that in *E. coli* [29] and *Yarrowia lipolytica* [30, 31]. Considering the inadequate resveratrol titer reported in *S. cerevisiae*, the construction of yeast strains with enhanced resveratrol production would lay a foundation for large-scale microbial production of resveratrol and other various high-value stilbenoids.

As reported, the biosynthesis of resveratrol as well as other *p*-coumaric acid-derived compounds in *S. cerevisiae* can be significantly improved by increasing the flux towards aromatic amino acid [32]. In most cases, only phenylalanine or tyrosine is used as a substrate owing to the substrate specificity of ammonia lyase employed in the biosynthetic pathway. The simultaneous utilization of phenylalanine and tyrosine has been preliminarily investigated by expressing a tyrosine ammonia lyase in the strain harboring the phenylalanine pathway, and enhanced *p*-coumaric acid production is achieved in spite of the low efficiency of tyrosine ammonia lyase [33]. Inspired by the synergistic effect between the phenylalanine pathway and tyrosine pathway in *p*-coumaric acid production, the combination of these two pathways is worthy of trial to make full use of intracellular tyrosine and phenylalanine. By searching for suitable ammonia lyases, a bi-functional phenylalanine/tyrosine ammonia lyase has been found to deaminate phenylalanine and tyrosine with similar catalytic efficiency, but it is usually applied as phenylalanine ammonia lyase or tyrosine ammonia lyase in biosynthetic pathways of natural products [34, 35]. Hence, further investigation in the application of the dual substrate specificity is attractive, especially in the construction of biosynthetic pathway for resveratrol and other *p*-coumaric acid-derived chemicals.

In this study, we constructed a resveratrol biosynthetic pathway incorporating both phenylalanine and tyrosine pathways in *S. cerevisiae* by employing a bi-functional phenylalanine/tyrosine ammonia lyase to generate the precursor *p*-coumaric acid. The copy numbers of partial pathway genes were increased via multiple rounds of integration. The precursor supply was also strengthened by overexpressing the upstream pathways for the precursors, including tyrosine, phenylalanine, and malonyl-CoA. Then the strain was further modified to produce resveratrol in minimal medium by enhancing the conversion of *p*-coumaric acid, and eliminating the amino acid addition in medium. The combinatorial engineering strategy enabled enhanced production of resveratrol in *S. cerevisiae*, with resveratrol titer reaching up to 4.1 ± 0.2 g/L via fed-batch fermentation.

## Materials and methods

### Strains and medium

*E. coli Trans 5α* (TransGen Biotech, Beijing, China) was used for propagation of plasmids. Luria–Bertani (LB) broth medium (5 g/L yeast extract, 10 g/L tryptone, 10 g/L NaCl) containing 50 mg/L of kanamycin was used to culture *E. coli* carrying transformed plasmids. *S. cerevisiae* strain BY4742 (*MATα*, *his3*Δ*1*, *leu2*Δ*0*, *lys2*Δ*0*, *ura3*Δ*0*) was used as the parent strain for resveratrol biosynthesis. YPD medium (10 g/L yeast extract, 20 g/L peptone, and 20 g/L glucose, also marked as YPD-20G when needed) was used for routine cultivation of *S. cerevisiae* strains. YPD medium with 40 g/L glucose was also used for fermentation in shake flasks and marked as YPD-40G. Geneticin (G418, 100 mg/L) was supplemented in the YPD agar plate for the selection of engineered yeast strains edited by CRISPR/Cas9. SC agar plates (synthetic complete drop-out medium, 20 g/L glucose, 6.7 g/L yeast nitrogen base without amino acids, and 0.8 g/L dropout powder minus appropriate amino acids) was used for the selection of engineered yeast strains edited by homologous recombination using HIS3, LEU2, URA3 and LYS2 respectively as selective markers. Minimal medium used in our study was based on studies described previously [36]. The medium contained 20 g/L or 40 g/L glucose, 15 g/L (NH_4_)_2_SO_4_, 8 g/L KH_2_PO_4_, 6.2 g/L MgSO_4_∙7H_2_O, 1.2% (*v/v*) vitamin solution, and 1% (*v/v*) trace metal solution. L-lysine (0.5 g/L) was added in minimal medium for the cultivation of strain BRT8 and BRT9. The trace metal solution contained 5.75 g/L ZnSO_4_∙7H_2_O, 0.32 g/L MnCl_2_∙4H_2_O, 0.47 g/L CoCl_2_∙6H_2_O, 0.48 g/L Na_2_MoO_4_∙2H_2_O, 2.9 g/L CaCl_2_∙2H_2_O, 2.8 g/L FeSO_4_∙7H_2_O and 80 mL 0.5 M EDTA, pH 8.0. The vitamin solution contained 0.05 g/L biotin, 1 g/L calcium pantothenate, 1 g/L nicotinic acid, 25 g/L *myo*-inositol, 1 g/L thiamine HCl, 1 g/L pyridoxal HCl and 0.2 g/L *p*-aminobenzoic acid.

### Genes and plasmids

For the construction of resveratrol-producing strains, *Pc4CL* (P14912.1) from *Petroselinum crispum*, *VvSTS* (P28343.2) from *Vitis vinifera*, *RtPAL/TAL* (P11544) from *Rhodotorula toruloides* (Synonyms *R. gracilis*), *AtCPR1* (NP_194183.1) and *AtC4H* (NP_180607.1) from *Arabidopsis thaliana* were codon-optimized for expression in *S. cerevisiae* and synthesized by Sangon Biotech (Shanghai, China). *SeACS*^*L641P*^ (MP052228.1) and selective marker cassettes (HIS3, LEU2 and URA3) were also synthesized by Sangon Biotech (Shanghai, China). Integration homologous arms (about 500 bp), *ScACC1*, *ScARO4*, *ScARO7,* and *ScARO2* were amplified from the genome of BY4742. *LYS2* including its homologous arms was amplified from the genome of W303-1A. *EcAROL* was amplified from the genome of *E. coli*. Mutants of *ACC1*, *ARO4* and *ARO7* were created by overlap PCR according to the previous report. Expression cassettes with promoters, terminators and genes were assembled to plasmid G418 using Minerva Super Fusion Cloning Kit (Yuheng Biotech, Suzhou, China). The plasmids pCas with specific *gRNA* sequences used for CRISPR/Cas9 editing were obtained according to the standard Quick-Change Site-Directed Mutagenesis protocol. The gRNA sequences of sites *cit2*, *lpp1*, *pha2,* and *dpp1* were designed using online tool E-CRISP [37], and the gRNA sequences of other sites were reported in the previous study [38]. Recombinant plasmids were confirmed by DNA sequencing. The detailed information of plasmids (Additional file 1: Table S2) and the primers (Additional file 1: Table S3) used in our study were listed in supplementary information.

### Construction of resveratrol-producing strains

Strains BR1, BRT2, BRT4 and BRT10 were constructed via homologous recombination using the HIS3, URA3, LEU2 and LYS2 markers respectively for auxotroph selection [39]. The linear DNA fragments of homologous arms, selective marker and expression cassette were transformed into yeast using the LiAc/SS carrier DNA/PEG method [40]. The resulting strains were selected on SC-His, SC-His-Ura, SC-His-Ura-Leu and SC-His-Ura-Leu-Lys agar plates respectively, and positive clones were verified by PCR amplification. Other strains were constructed via CRISPR/Cas9 gene editing system [38]. The specific plasmid pCas and the linear DNA fragments with homologous arms and expression cassette were transformed into yeast using the LiAc/SS carrier DNA/PEG method [40], and the resulting strains were selected on YPD agar plate containing G418 (100 mg/L). The recombinant strains were validated by PCR amplification, and then the specific plasmid pCas was eliminated by dilution and cultured in fresh YPD medium.

The detailed information of the yeast strains used or constructed in this study is shown in Table 1.

**Table 1:** Strains used in this study

Strains	Genotype/Description	References
BY4742	*MATα*, *his3*Δ*1*, *leu2*Δ*0*, *lys2*Δ*0*, *ura3*Δ*0*	[41]
BR1	BY4742-*yorw∆17*::HIS3/*T*_*ADH1*_-*VvSTS*-* P*_*PGK1*_/*P*_*TEF1*_-*Pc4CL*-*T*_*CYC1*_	This study
BRT2	BR1-*ura3*::* T*_*ADH1*_-*RtPAL/TAL*-* P*_*PGK1*_/*P*_*TDH3*_-*VvSTS*-* T*_*CYC1*_/ URA3	This study
BRT3	BRT2*- cit2∆*::* T*_*ADH1*_-*RtPAL/TAL*-* P*_*PGK1*_/*P*_*TDH3*_-*VvSTS*-* T*_*CYC1*_	This study
BRT3-1	BRT2*- pha2∆*	This study
BRT4	BRT3- *leu2*::* T*_*ADH1*_- *AtCPR1*-* P*_*PGK1*_/*P*_*TDH3*_-*AtC4H- T*_*CYC1*_/ LEU2	This study
BRT5	BRT4- *lpp1∆*::* T*_*ADH1*_-*RtPAL/TAL*-* P*_*PGK1*_/*P*_*TDH3*_-*VvSTS*-* T*_*CYC1*_	This study
BRT6	BRT5*-1309a*::* T*_*ADH1*_-*ARO4 *^*K229L*^—*P*_*PGK1*_/*P*_*TEF1*_-*ARO7 *^*G141S*^—*T*_*CYC1*_	This study
BRT7	BRT6*-511b*::*T*_*ADH1*_-*EcAROL*—*P*_*PGK1*_/*P*_*TEF1*_-*ARO2*—*T*_*CYC1*_	This study
BRT8	BRT7*-pdc6∆*::* P*_*TEF1*_-*ScACC1*^*S659A/S1157A*^ -*T*_*CYC1*_	This study
BRT8-1	BRT7*-911b*::* T*_*ADH1*_-*SeACS *^*L641P*^ -*P*_*TDH3*_	This study
BRT9	BRT8-*dpp1∆*::* T*_*ADH1*_-*VvSTS*-* P*_*PGK1*_/* P*_*TEF1*_-*Pc4CL*-* T*_*CYC1*_	This study
BRT10	BRT9-*lys2*::LYS2	This study

### Strains cultivated in shaking flasks

Single colonies were picked from the YPD agar plate, inoculated into 5 mL YPD in culture tubes, and incubated overnight at 30 °C in a rotary shaker (200 rpm). The seed cultures were then transferred into 50 mL fresh medium to reach an initial OD_600_ of 0.1–0.2 and grown under the same conditions. The fermentation ended at 72 h and 144 h respectively when strains cultured in YPD containing 20 g/L glucose (YPD-20G) and 40 g/L glucose (YPD-40G) to compare the performance of different strains. Time courses were plotted every 12 h or 24 h until resveratrol titer reached a plateau. Cell growth was evaluated by OD_600_ using a Shimadzu UV-1900i spectrophotometer, and dry cell weight (DCW) in shake flasks was calculated using the coefficient, 1 OD_600_ = 0.3722 g/L DCW.

## Analysis of the metabolites

For quantification of *p*-coumaric acid and resveratrol, 200 μL of culture broth was mixed thoroughly with 600 μL of ethanol. The supernatant of the mixture was collected by centrifugation at 12,000* g* for 10 min and then filtered through 0.22 μm filters. The samples were analyzed on an HPLC (SHIMADZU LC20A) system equipped with Kinetex 5 µm C18 100 Å LC Column (250 × 4.6 mm). The gradient program was performed with solvent A (water containing 0.1% formic acid) and solvent B (acetonitrile) as mobile phase. The program started with 95% of solvent A and 5% of solvent B, and the concentration of solvent B was increased to 10% linearly within 3 min, continued up to 30% at 5 min, up to 70% at 10 min and finally up to 95% at 10.5 min, then kept to 12.5 min, finally the concentration of solvent B was decreased to 5% at 13 min and kept until 16 min. The flow rate was 1 mL/min with a constant column temperature at 30 °C. Resveratrol and *p*-coumaric acid were detected with a wavelength of 303 nm.

The concentrations of glucose and ethanol in culture broth were measured by a biosensor (SBA-40D, Shandong, China) using supernatant of the culture broth collected by centrifugation at 12,000* g* for 10 min.

### Fed-batch fermentation

Fed-batch fermentation for resveratrol production was conducted in a 3-L bioreactor (Baoxing, Shanghai, China) using minimal medium with approximately 20 g/L glucose. Seed solution (150 mL) cultured in YPD-20G at 30 °C and 200 rpm for 18 h was inoculated into 1.5 L minimal medium. Fermentation was carried out at 30 °C and pH was controlled at 5.0 by adding ammonium hydroxide and phosphoric acid automatically. Dissolved oxygen (DO) was maintained at 30% by adjusting stirring rate with a constant air flow rate of 1 *vvm*. Glucose (750 g/L) was fed with a feeding rate ranged from 1–3 g/L∙h. Additional sterile defoamer was manually added to the reactors when foaming was observed. Sampling was carried out three times per day to measure OD_600_, dry cell weight, glucose, ethanol, *p*-coumaric acid and resveratrol.

## Results

### Effect of the bi-functional phenylalanine/tyrosine ammonia lyase on de novo resveratrol production

**Fig. 2:**
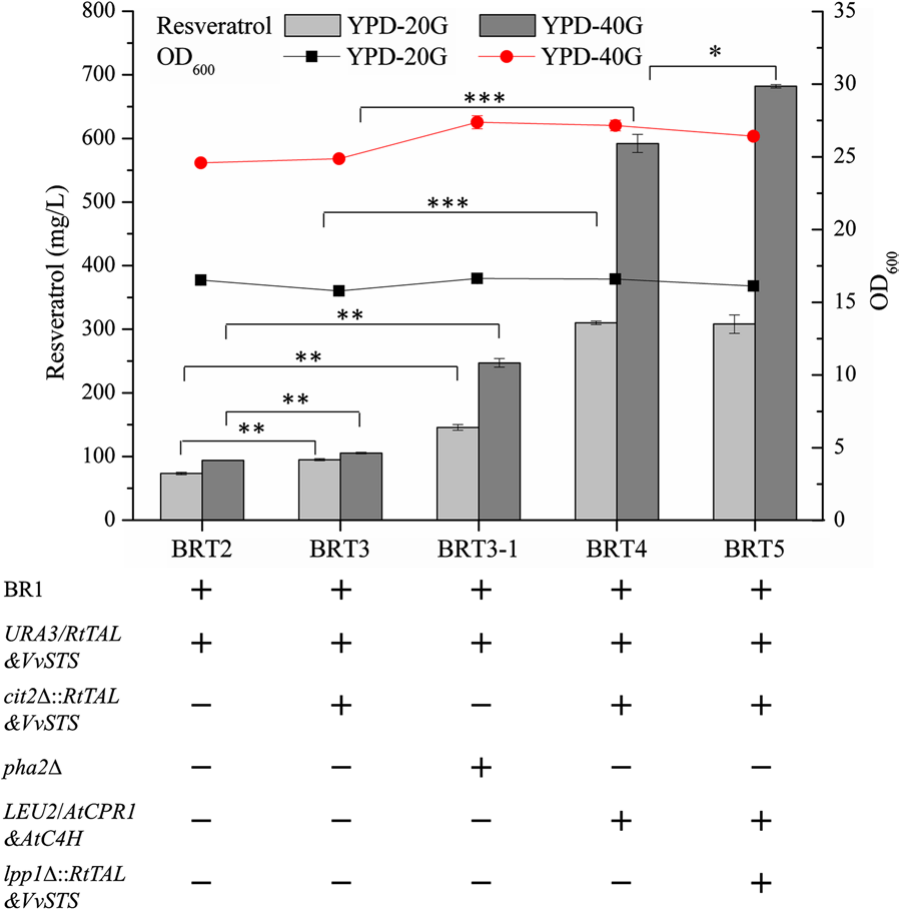
Resveratrol production and cell density of engineered strains obtained by pathway engineering via both phenylalanine pathway and tyrosine pathway using bi-functional phenylalanine/tyrosine ammonia lyase. The resveratrol titer was measured after the strains cultured in shake flasks using YPD medium with 20 g/L glucose (YPD-20G) for 72 h and 40 g/L glucose (YPD-40G) for 144 h respectively. Statistical analysis was performed by using Student’s *t-test* (two-tailed; two-sample assuming equal variance; *p < 0.05, **p < 0.01, ***p < 0.01) focused on the resveratrol production. The displayed average values and standard deviations were calculated from three independent biological experiments

In the present study, *p*-coumary-CoA ligase from *P. crispum* (*Pc4CL*) and resveratrol synthase from *V. vinifera* (*VvSTS*) were firstly integrated into the *yorw∆17* genomic loci of strain BY4742, and the resulting strain BR1 produced 3.7 mg/L resveratrol in YPD medium containing 0.5 mmol/L *p*-coumaric acid as a precursor, indicating the functional expression of *Pc4CL* and *VvSTS* (Additional file 1: Figure S1). Then, a bi-functional phenylalanine/tyrosine ammonia-lyase from *R. toruloides* (*RtPAL/TAL*) together with another copy of *VvSTS* was integrated into the *ura3* site of strain BR1. The resulting strain BRT2 enabled the de novo production of resveratrol via tyrosine pathway and produced 73.4 mg/L resveratrol in YPD-20G medium without any precursor addition. To enhance the resveratrol production via only tyrosine pathway, strain BRT2 was further modified by integrating another copy of *RtPAL/TAL* and *VvSTS* into the *cit2* site to generate BRT3. The strain BRT3 resulted in a 29% improvement in resveratrol production at 95.1 mg/L in YPD-20G medium. Also, the synthesis of phenylalanine in strain BRT2 was blocked by deleting *PHA2* to generate BRT3-1, leading to a 99% and 163% improvement of resveratrol production in YPD-20G and YPD-40G, respectively (Fig. 2).

To make the best of the dual substrate specificity of *RtPAL/TAL*, *At*C4H and *At*CPR1 converting the cinnamic acid into *p*-coumaric acid was introduced to strain BRT3, and the resulting strain BRT4 obtained a 226% and a 462% improvement in resveratrol production when cultured in YPD-20G and YPD-40G respectively (Fig. 2). This result demonstrated that tapping on both phenylalanine and tyrosine pathways proved to be more effective than the tyrosine pathway alone in resveratrol production. As no cinnamic acid or *p*-coumaric acid was observed at the end of fermentation of strain BRT4, one more copy of *RtPAL/TAL* and *VvSTS* was integrated into the site *lpp1* to obtain strain BRT5. Strain BRT5 showed a similar resveratrol titer when cultured in YPD-20G, but achieved a 15% increase in resveratrol titer when cultured in YPD-40G, with a titer of 308.2 mg/L and 682.0 mg/L respectively (Fig. 2).

### Enhancement of precursor supply for further improvement of the resveratrol production

**Fig. 3:**
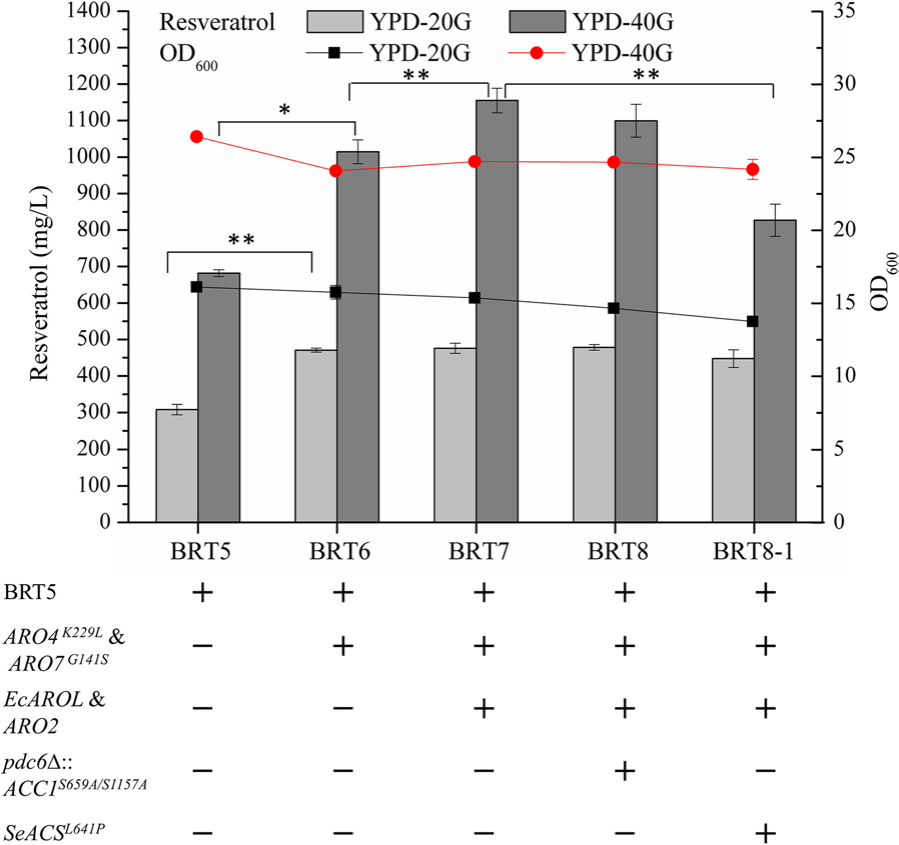
Resveratrol production and cell density of engineered strains obtained by increasing the precursor supply. The resveratrol titer was measured after the strains were cultured in shake flasks using YPD medium with 20 g/L glucose (YPD-20G) for 72 h and 40 g/L glucose (YPD-40G) for 144 h respectively. Statistical analysis was performed by using Student’s *t-test* (two-tailed; two-sample assming equal variance; *p < 0.05, **p < 0.01 and signs were not shown when *p* > 0.05) focused on the resveratrol production. The displayed average values and standard deviations were calculated from three independent biological experiments

To further increase the resveratrol titer, the supply of precursors including aromatic amino acids and malonyl-CoA were enhanced in our study. Firstly, the feedback-inhibition resistant versions of DAHP synthase (ARO4^K229L^) and chorismate mutase (ARO7^G141S^) were integrated into the *1309a* site of strain BRT5, and resulted in a 50% improvement of resveratrol titer in batch cultures (Fig. 3). Then, the effect of shikimate kinase AROL from *E. coli* and chorismate synthase (ARO2) were tested by integrating into strain BRT6, the resulting strain BRT7 produced almost the same amount of resveratrol when cultured in YPD-20G, but produced a 14% higher titer of resveratrol when cultured in YPD-40G (Fig. 3).

To test the effect of increasing intracellular malonyl-CoA in yeast on resveratrol titer, strain BRT7 was modified and evaluated by overexpressing the acetyl-CoA carboxylase mutant ACC1^S659A/S1157A^ or the acetyl-CoA synthase (*Se*ACS^L641P^) derived from *S. enterica*. The results showed that the introduction of ACC1^S659A/S1157A^ did not show improvement of resveratrol production when cultured in YPD medium, and the introduction of *Se*ACS^L641P^ led to a 30% decrease of resveratrol titer in YPD-40G (Fig. 3). These results indicated that the malonyl-CoA availability might not be the bottleneck in resveratrol production in *S. cerevisiae* under the current status, mainly due to the regulation networks of yeast.

The time courses of strains BRT2, BRT5 and BRT8 were monitored, and the results showed that extracellular glucose could not be detected after 24 h of cultivation, while the biomass and resveratrol production continued to increase after glucose depletion. The concentration of ethanol and *p*-coumaric acid reached the highest and then decreased slowly until exhaustion at the end of fermentation (Additional file 1: Figure S2). The cell growth, glucose and ethanol consumption decreased, but the accumulated *p*-coumaric acid increased as the yeast was modified, thus strain BRT8 took about 156 h to reach the highest resveratrol titer. No apparent *p*-coumaric acid or cinnamic acid was detected at the end of the fermentation in any of the strains that were capable of de novo resveratrol production. The highest titer of resveratrol was obtained in strain BRT8 (478.5 ± 7.6 mg/L) and strain BRT7 (1155.0 ± 14.3 mg/L) when cultured in flasks using YPD-20G and YPD-40G respectively.

### Strains tailor-made for resveratrol production in minimal medium

The performance of strain BRT8 cultured in complex medium and minimal medium were tested and compared by monitoring the time courses. The results showed that glucose consumption and ethanol accumulation in minimal medium were much slower than that in YPD-20G medium, as glucose was not detected after 36 h, and the accumulated ethanol was only partially consumed after 120 h. The accumulated *p*-coumaric acid during the fermentation in minimal medium was also lower than that in YPD medium, as well as its consumption. The cell density and resveratrol titer were also much lower than that in YPD-20G, with a maximum resveratrol titer of 256.2 mg/L (Additional file 1: Figure S3). However, the resveratrol yield over biomass (the ratio between the concentrations of resveratrol produced and dry cell weight, mg resveratrol per g of dry cell weight) was calculated and compared, and the results demonstrated that resveratrol yield in minimal medium was higher than in YPD medium throughout the fermentation, especially at the early stage of the fermentation (Additional file 1: Figure S4). These results suggested that the cost-efficient minimal medium could be more attractive in high-cell density fermentation for resveratrol production.

**Fig. 4:**
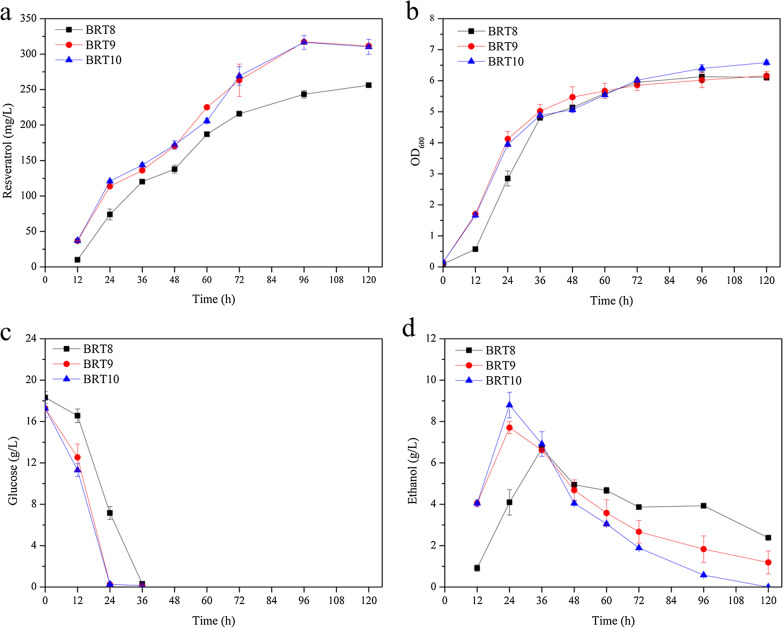
Time courses of **a** resveratrol production, **b** cell growth, **c** glucose consumption and **d** ethanol concentration of strains BRT8, BRT9 and BRT10 cultured in minimal medium. 0.5 g/L lysine was added in the minimal medium for the growth of strain BRT8 and BRT9. Error bars represent standard deviations from three independent biological experiments

As shown in Additional file 1: Figures S2 and S3, some *p*-coumaric acid accumulated during the fermentation of strain BRT8. To enhance the conversion of *p*-coumaric acid during fermentation, the expression cassette of *Pc4CL* and *VvSTS* was integrated into the *dpp1* site of strain BRT8 to generate strain BRT9. The cell density and resveratrol titer of strain BRT9 in YPD medium were not significantly different from that of strain BRT8 (Additional file 1: Table S4). Time courses in minimal medium were also monitored and the results showed that strain BRT9 performed better than strain BRT8 in glucose and ethanol consumption, cell growth, resveratrol production (Fig. 4) and the conversion of *p*-coumaric acid (Additional file 1: Figure S5). This probably caused by the higher conversion rate of *p*-coumaric acid catalyzed by the additional *Pc*4CL, which consumed ATP and CoA to facilitate the metabolism of the engineering strain. As a result, strain BRT9 produced 317.2 ± 1.9 mg/L of resveratrol after 96 h of fermentation, which was 23.8% higher than that of strain BRT8.

For the auxotrophic yeast strain BY4742 used in the study, HIS3, URA3 and LEU2 were used as the selective markers in the construction of recombinant strains, histidine, leucine and uracil are no longer essential for cell growth. Inspired by the process of marker recovery, LYS2 in strain BRT9 was also recovered to obtain strain BRT10. This strain was non-auxotrophic and grown in minimal medium without exogenous amino acids. The results showed that strain BRT10 performed similarly to strain BRT9 in resveratrol production, cell growth, glucose consumption, ethanol and *p*-coumaric acid concentration in both YPD medium (Additional file 1: Table S4) and minimal medium (Fig. 4), indicating costly lysine in minimal medium can be avoided by the recovery of LYS2.

### Efficient production of resveratrol in a 3-L bioreactor by fed-batch fermentation

**Fig. 5:**
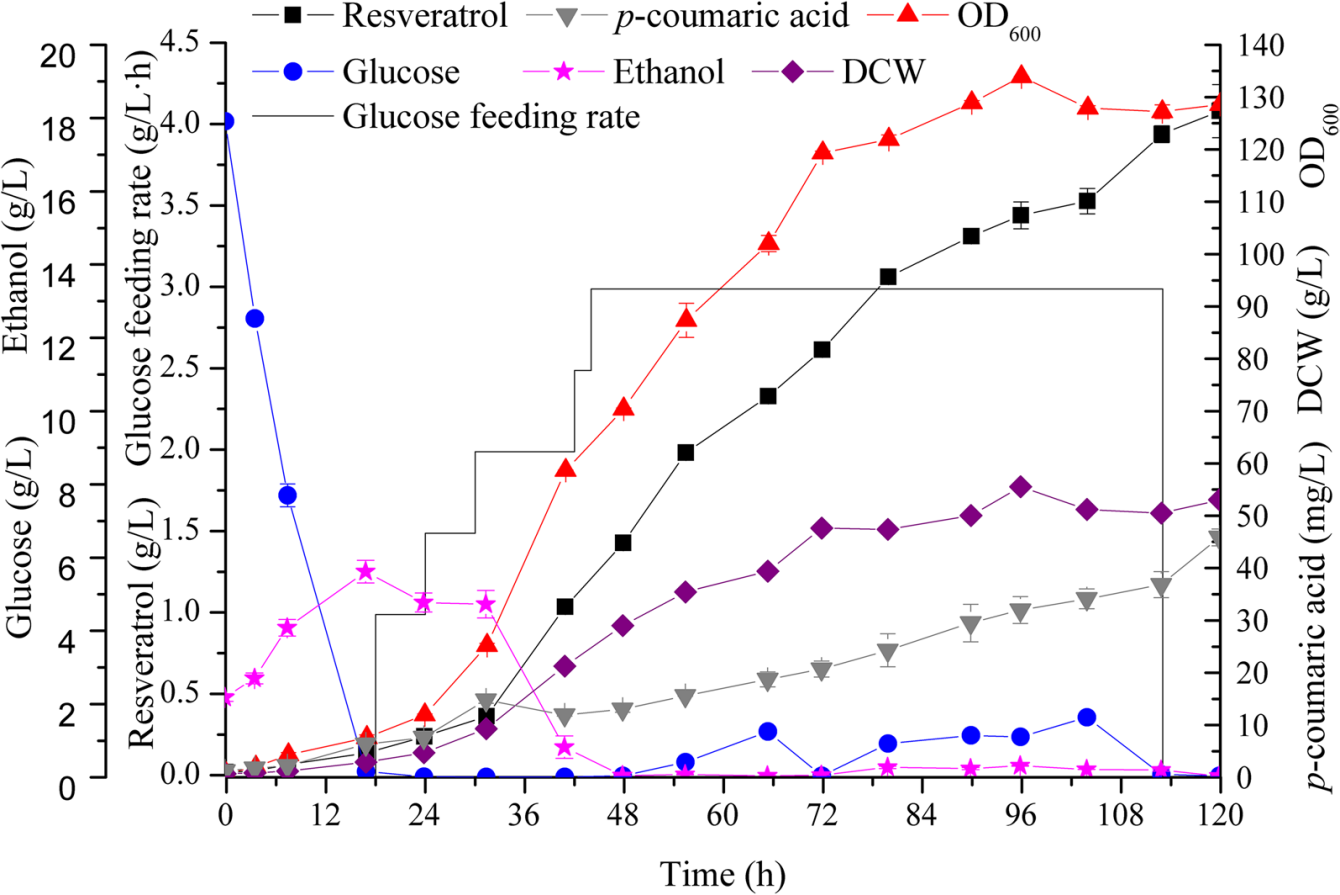
Fed-batch fermentation in a 3-L bioreactor of the non-auxotrophic strain BRT10 using minimal medium without adding any amino acids. DCW: dry cell weight. Error bars represent standard deviations from three detections of each sample

To further promote resveratrol production and evaluate the performance of the non-auxotrophic strain in minimal medium without amino acids supply in a scale-up condition, we performed fed-batch fermentation in a 3-L bioreactor. As shown in Fig. 5, about 18 g/L glucose in the initial minimal medium was consumed within 17 h, and the ethanol was accumulated to 5.6 g/L and then used as the carbon source. The feeding of glucose was initiated at 18 h with a relatively low rate of 1 g/L∙h, because DO became relatively stable (Additional file 1: Figure S6), and ethanol also acted as a carbon source. During the fermentation, the glucose feeding rate was increased based on the detected glucose concentration, ethanol concentration as well as DO fluctuation. Once the initial glucose was consumed, it could not be detected in the fermentation broth until the feeding rate increased to 3 g/L∙h. At this feeding rate, the highest glucose concentration was just 1.6 g/L, suggesting that glucose did not accumulate at the fermentation stage. The concentration of ethanol was lower than 0.3 g/L and stopped accumulating after 48 h, probably due to the restricted feeding of glucose. The glucose feeding was stopped at 113 h when OD_600_ reached a plateau. The concentration of *p*-coumaric acid was accumulated slowly during the fermentation to a titer of 45.5 mg/L. A fast-growing phase and a fast resveratrol accumulation were observed between 24 and 72 h, while DO fluctuated around 30% by automatically adjusting the stirring rate (Additional file 1: Figure S6). At the end of the fed-batch fermentation, 4.1 ± 0.2 g/L resveratrol and 53.1 ± 0.1 g/L dry cell weight were obtained from feeding about 250 g/L glucose. This result demonstrated that minimal medium without lysine supply could be used in the scale-up fed-batch fermentation of engineered yeast strains.

## Discussion

Previous studies have synthesized resveratrol in engineered yeasts from precursors (*p*-coumaric acid, L-phenylalanine or L-tyrosine) or low-cost materials such as glucose, ethanol, lactose-rich waste or wine wastes [23, 26–28]. In *S. cerevisiae*, de novo resveratrol productions were achieved via either tyrosine pathway or phenylalanine pathway [23–28], while in *Y. lipolytica*, resveratrol titers were improved via both phenylalanine pathway and tyrosine pathway by simultaneously expressing phenylalanine ammonia lyase and tyrosine ammonia lyase [31, 42]. A similar strategy was tested in *S. cerevisiae* for *p*-coumaric acid production, but the low efficiency of tyrosine ammonia lyase limited the synergistic effect from the combination of the two pathways [33]. To make full use of intracellular phenylalanine and tyrosine, a bi-functional phenylalanine/tyrosine ammonia lyase *RtPAL/TAL*, which shows Km-values for phenylalanine and tyrosine in the same order of magnitude, would be attractive to enable the efficient transformation of both phenylalanine and tyrosine [43].

In previous reports, the bi-functional phenylalanine/tyrosine ammonia lyase *RtPAL/TAL* was mainly used to produce different compounds from precursor phenylalanine or tyrosine without the expression of cinnamate-4-hydroxylase [34, 44, 45]. For resveratrol production, this bi-functional property was preliminarily studied by adding tyrosine as a precursor, and improved resveratrol titer was obtained. Nevertheless, the titer was fairly low (5.8 mg/L) and the strain was genetically unstable as genes were expressed in several episomal plasmids [46]. In our study, we first compared the effect of *RtPAL/TAL* on resveratrol titer with or without the integration of cinnamate-4-hydroxylase, and demonstrated that resveratrol titer could be significantly increased by combining both tyrosine pathway and phenylalanine pathway using *RtPAL/TAL*. We believed that replacing phenylalanine ammonia lyase with the bi-functional phenylalanine/tyrosine ammonia lyase or introducing the bi-functional enzyme into the strains that harbored the phenylalanine pathway would be a promising strategy to improve the metabolic flux into *p*-coumaric acid and its derived compounds.

In order to construct an efficient cell factory for resveratrol production, strategies proved to be effective in the production of resveratrol as well as other compounds sharing the same precursor were applied in the present study. These strategies, including the multi-copy integration of biosynthetic pathway genes, overexpression of pathway genes to improve metabolic flux to aromatic amino acids and malonyl-CoA, and knockouts of by-pathway genes, showed a positive effect on resveratrol production [12]. With these approaches, the resveratrol titer in the YPD medium reached 1155.0 ± 14.3 mg/L in batch fermentation cultured in shake flasks (Fig. 3), which exceeded the highest titer reported in *S. cerevisiae* previously [25]. Considering the biomass and resveratrol titer in the YPD medium were much higher than those in the simple medium [47], our strain was also tested and tailor-made for growth in minimal medium, as a higher resveratrol yield over biomass (Additional file 1: Figure S4) was obtained and attractive in scale-up fermentation.

As a key cell factory already used for the production of a wide range of products, many *S. cerevisiae* strains used as starting hosts are harboring various auxotrophic genotypes [48]. In most cases, the disrupted genes such as *ura3*, *leu2*, *his3*, and *trp1* in the starting strains were recovered and used as selection markers for genetic manipulations, but disrupted *lys2* in strains such as BY4742 is not yet recovered, thus lysine is indispensable in the minimal medium for cell growth [49–51]. In this study, we first *in-situ* restored the *lys2* gene in the engineered strain and found that the *lys2* recovery could fully eliminate the dependence on lysine in minimal medium (Fig. 4). This simple restoration in genome would cut down the cost of lysine as well as simplify the operation process.

To our knowledge, resveratrol production by our non-auxotrophic strain BRT10 represented the highest resveratrol titer in engineered *S. cerevisiae*, which was increased by fivefold compared with that of the strain ST4990 constructed by Li et al. [25]. In the present study, the dry cell weight and the resveratrol yield on glucose were about 2.4 times and 1.7 times higher than that of the reported strain ST4990 respectively [25], demonstrating that improvement in both cell density and strain performance contributed to enhanced resveratrol production. Although a higher cell density was achieved in our fed-batch fermentation, the dry cell weight (53.1 ± 0.1 g/L) was still much lower than that of other engineered *S. cerevisiae* (higher than 120 g/L) [50]. Moreover, the resveratrol yield in fed-batch fermentation was decreased compared with the batch fermentation in shake flasks (Fig. 4), showing that there is great potential to optimize the fed-batch fermentation process. Besides, the resveratrol yield on glucose was still much lower than the maximum theoretical yield (355 mg/g glucose) [52], thus, strategies to further improve the strain performance would be promising.

## Conclusions

In this study, efficient resveratrol production (4.1 g/L) in engineered yeast *S. cerevisiae* was realized via combining strategies, including the introduction of a bi-functional phenylalanine/tyrosine ammonia lyase into the resveratrol biosynthetic pathways, enhancement of precursors supply, overexpression of rate-limiting enzymes, recovery of auxotrophic selection markers, and a scale-up of fed-batch fermentation. This work highlights the benefit of combining the phenylalanine and tyrosine pathways of resveratrol biosynthesis by one enzyme and demonstrates the advantage of eliminating the lysine addition in fed-batch fermentation in resveratrol biosynthesis. This study will accelerate the development of *S. cerevisiae* strains capable of producing high-value resveratrol as well as other *p*-coumaric acid-derived products.

## Supplementary Information

**Additional file 1: Table S1**. Resveratrol production in various hosts. **Table S2**. Plasmids used in this study. **Table S3**. Primers used in this study. **Table S4**. Cell densities and resveratrol titers of strains BRT9 and BRT10 cultured in YPD medium. **Figure S1.** Production of resveratrol in engineered strain BR1. Standards of *p*-coumaric acid and resveratrol, and samples of strain BR4742 and BR1 were analyzed by HPLC. Standards were dissolved in methanol, and fermentative broths of strain BY4742 and BR1 were mixed with ethanol. Strains were cultured in YPD medium with 0.5 mmol/L *p*-coumaric acid for 72 h. **Figure S2.** Time courses of (a) resveratrol production, (b) cell growth, (c) glucose consumption, (d) ethanol concentration and (e) *p*-coumaric acid concentration of strains BRT2, BRT5 and BRT8 cultured in YPD medium with 20 g/L and 40 g/L glucose respectively. Error bars represent standard deviations from three independent biological experiments. **Figure S3.** Time courses of (a) resveratrol production, (b) cell growth, (c) glucose consumption, (d) ethanol concentration and (e) *p*-coumaric acid concentration of strain BRT8 cultured in different media. Error bars represent standard deviations from three independent biological experiments. **Figure S4.** Time course of resveratrol yield on dry cell weight when strain BRT8 was cultured in YPD or minimal medium. DCW: dry cell weight. Error bars represent standard deviations from three independent biological experiments. **Figure S5.** Time course of *p*-coumaric acid concentration of strains BRT8, BRT9 and BRT10 cultured in minimal medium. 0.5 g/L lysine was added in the minimal medium for the growth of strain BRT8 and BRT9. Error bars represent standard deviations from three independent biological experiments. **Figure S6.** Parameters during the fed-batch fermentation in the bioreactor using the non-auxotrophic strain BRT10 in minimal medium.

## Data Availability

All data generated or analyzed during this study are included in this published article and its additional files.
