# Supplementary material for: Efficient biosynthesis of resveratrol via combining phenylalanine and tyrosine pathways in Saccharomyces cerevisiae
Source: Microb Cell Fact. 2023 Mar 8;22:46. doi: 10.1186/s12934-023-02055-9 (PMC9996981; doi:10.1186/s12934-023-02055-9)
Supplement: Supplementary file 1 — Additional file 1: Table S1. Resveratrol production in various hosts. Table S2. Plasmids used in this study. Table S3. Primers used in this study. Table S4. Cell densities and resveratrol titers of strains BRT9 and BRT10 cultured in YPD medium. Figure S1. Production of resveratrol in engineered strain BR1. Standards of p-coumaric acid and resveratrol, and samples of strain BR4742 and BR1 were analyzed by HPLC. Standards were dissolved in methanol, and fermentative broths of strain BY4742 and BR1 were mixed with ethanol. Strains were cultured in YPD medium with 0.5 mmol/L p-coumaric acid for 72 h. Figure S2. Time courses of (a) resveratrol production, (b) cell growth, (c) glucose consumption, (d) ethanol concentration and (e) p-coumaric acid concentration of strains BRT2, BRT5 and BRT8 cultured in YPD medium with 20 g/L and 40 g/L glucose respectively. Error bars represent standard deviations from three independent biological experiments. Figure S3. Time courses of (a) resveratrol production, (b) cell growth, (c) glucose consumption, (d) ethanol concentration and (e) p-coumaric acid concentration of strain BRT8 cultured in different media. Error bars represent standard deviations from three independent biological experiments. Figure S4. Time course of resveratrol yield on dry cell weight when strain BRT8 was cultured in YPD or minimal medium. DCW: dry cell weight. Error bars represent standard deviations from three independent biological experiments. Figure S5. Time course of p-coumaric acid concentration of strains BRT8, BRT9 and BRT10 cultured in minimal medium. 0.5 g/L lysine was added in the minimal medium for the growth of strain BRT8 and BRT9. Error bars represent standard deviations from three independent biological experiments. Figure S6. Parameters during the fed-batch fermentation in the bioreactor using the non-auxotrophic strain BRT10 in minimal medium. [file 12934_2023_2055_MOESM1_ESM.pdf]

Supplementary Information

Efficient biosynthesis of resveratrol via combining phenylalanine and tyrosine pathways in *Saccharomyces cerevisiae*

Lijun Meng*, Mengxue Diao, Qingyan Wang, Longyun Peng, Jianxiu Li, Nengzhong Xie*

State Key Laboratory of NonFood Biomass and Enzyme Technology, National Engineering Research Center for Non-Food Biorefinery, Guangxi Biomass Engineering Technology Research Center, Guangxi Key Laboratory of Bio-refinery, Guangxi Academy of Sciences, 98 Daling Road, Nanning, 530007, China

***Corresponding Authors**

Lijun Meng, E-mail: menglj@gxas.cn; Phone: +86-0771-2503356

Nengzhong Xie, Email: xienengzhong@gxas.cn; Phone: +86-0771-2503942.

Supplementary Tables

Table S1. Resveratrol production in various hosts [a]

Strains (Parent strains)	Related gene cassettes	Notes	Carbon source	Resveratrol (mg/L)	Scale	References
E. coli						
S4 [c] [*E. coli* BL21 (DE3)]	*pCDFDuet-Pc4CL2-VvSTS; pIBR-181-ScmatBC*	Modular pathway engineering and the introduction of malonate assimilation pathway genes	Glucose	151	Flask (Batch)	[1]
N.A.[b c] [*E. coli* BL21 (DE3)]	*LacZ::EcaroGfbr, EctyrAfbr,* pCDFDuet-*TcTAL(Variants)-Pc4CL,* pETDuet-*VvSTS,* pACYC-*RtmatC-RtmatB-dCas9,* pCOLA-*fabF(high)/fabB(medium)/fabI(low)/fabH(low)*	Introduction of malonate assimilation pathway, Down regualtion of fatty acid biosynthesis pathway genes, Optimization the expression of *TcTAL* based on mRNA structure	Glucose	304.5	Flask (Batch)	[2]
N.A. [c] [*E. coli* BL21 (DE3)]	pET28a-*Vvsts*, pCDFDuet-1-*Setal*, pACYCDuet-1-*At4cl*	Construction of a new probabilistic computational model to simulate the microbial production of resveratrol	Glucose	172.8	Flask (Batch)	[3]
N.A. [*E. coli* BW25113 (DE3)]	*ΔtyrR ::VvSTS, ΔtrpED::RgTAL-Pc4CL*	First utilized the site-specific integration strategy to produce resveratrol in *Escherichia coli*	Glucose	4.6	Flask (Batch)	[4]

**Unconventional yeast**						
T4V-ARO47m [c]<br>(*P. pastoris*)	CBS7435 *Δdnl4 Δhis4*<br>pPGP-*HaTAL-At4CL-VvVST*,<br>pPGPH-*ARO4*^{K229L}-*ARO7*^{G141S} [G418^{r}, Hyg^{r}]	Development of an L-Tyr chassis in *Pichia pastoristo* produce various aromatic secondary metabolites	Glycerol	451<br><br>1825	Flask<br>(Batch)<br>Bioreactor<br>(Batch)	[5]
Ss-T4V-aro7m<br>(*S. stipites*)	*ΔURA5, ΔADE2*<br>*ADE2: PIR1p-HaTAL1-TEF1t, ENO1p-At4CL2-UAGt, TEF1p-VvVST1-GLN1t*<br>*URA5: ENO1p-SsARO7*^{G139S}*-UAGt*	Production of resveratrol from a wide range of sugars as carbon sources by a using the yeast *Scheffersomyces stipitis*	Glucose<br>Cellobiose<br>Sucrose	238<br>530<br>669	Flask<br>(Batch)	[6]
Y. lipolytica						
T2P2<br>(*Y. lipolytica* Pold)	*pTEF-FjTAL x2*,<br>*pTEF-PAL x2, pTEF-C4H x2, pTEF-4CL1 x2*,<br>*pTEF-VvVST x2*, Ura^{+}, Leu^{+}	Resveratrol production from glycerol, Multi-copy integration of pathway genes	Glycerol	430	Bioreactor<br>(Batch)	[7]
N.A.<br>(*Y. lipolytica* Po1fk)	Δ*ylTYR1*, Δ*ylTRP2*, Δ*ylTRP3*, Δ*ylARO8*, Δ*ylARO9*, Δ*ylPYK*, Δ*ylPHA2*<br>*ylARO1, ylARO2, ylARO3, ylARO4, ylARO5, scARO4*^{K229L}, *aroG*^{S180F}*, ylTKT, bbxfpK, acxpk, rgTAL ylTYR1, vvSTS1, pc4CL2::Leu*	Mainly focus on the engineering the chassis for *de novo* synthesis of five aromatic-derived natural products and chemicals	Glucose	12.67	Flask<br>(Batch)	[8]
ST9671<br>(*Y. lipolytica* W29)	*ku70Δ::SpCas9-EcDsdAMX4*<br>*IntC2::PrTEFintron-FjTAL-TLip2*<br>*IntC3::TPex20-At4CL1-PrGPD-PrTEFintron-VvVST1-TLip2*<br>*IntE1::TPex20-YlARO7*^{G139S}*-PrGPD-PrTEFintron-YlARO4*^{K221L}*-TLip2*<br>*IntE4,D1,E3,F3,A1::TPex20-FjTAL-PrTEFintron-TPex20-At4CL1-PrGPD-PrTEFintron-VvVST1-TLip2*	Integration of six copies of *FjTAL*, *At4CL1*, *VvVST1*; Significantly increase of resveratrol titer	Glucose	409<br><br>12400	Flask<br>(Batch)<br>Bioreactor<br>(Fed-batch)	[9]

ST890<br>(*Y. lipolytica*<br>Po1fk)	D17::P_{TEF}-*FjTAL*-T_{XPR2}<br>F1-3::P_{TEF}-*ARO4*^{K221L}-T_{XPR2}-P_{TEF}-*ARO7*^{G139S}-T_{XPR2}-<br>P_{TEF}-*ARO1*-T_{XPR2}<br>C3::P_{TEF}-*AtPAL*-T_{XPR2}-P_{TEF}-*AtC4H*-T_{XPR2}-P_{TEF}-*AtATR2*-T_{XPR2}<br>E1::P_{TEF}- *YlCYB5*-T_{XPR2}-P_{TEF}-*Aro3*^{K225L}-T_{XPR2}<br>AXP::P_{TEF}-*CaFPK*-T_{XPR2}-P_{TEF}-*BsPTA*-T_{XPR2}<br>26S rDNA::$P_{8xUASTEFin}$-*Pc4CL1-EAAAK-VvSTS*-T_{XPR2}-<br>P_{LEU}-*URA3*-T_{XPR2}<br>ZETA::$P_{8xUASTEFin}$-*Pc4CL1-EAAAK-VvSTS*-T_{XPR2}-<br>P_{LEU}-*LEU*-T_{XPR2}<br>△DGA1	Multi-copy integration of *Pc4CL-EAAAK-VvSTS* and screening, Morphology control in Fed-batch fermentation	Glucose	819<br><br>22500	Flask<br>(Batch)<br>Bioreactor<br>(Fed-batch)Flask	[10]
S. cerevisiae						
ST4152<br>(*S. cerevisiae*<br>CEN.PK102-5B)	Ty-(P_{TEF1}-*HaTAL*, P_{PGK1}-*At4CL1*, P_{TEF1}-*VvVST1*),<br>P_{TEF1}-*ScARO7*^{G141S}, P_{PGK1}-*ScARO4*^{K229L},<br>P_{TEF1}-*ScACC1*^{S659A,S1157A}	Multi-copy integration of *HaTAL-At4CL1-VvSTS1*	Glucose<br><br>Ethanol	415.6<br><br>531.4	Bioreactor<br>(Fed-batch)	[11]
ST4992<br>(*S. cerevisiae*<br>CEN.PK102-5B)	P_{TEF1}-*AtATR2*, P_{PGK1}-*CYB5*, P_{TEF1}-*VvVST1*)<br>Ty-(P_{TDH3}-*AtPAL2*, P_{FBA1}-*AtC4H*, P_{PGK1}-*At4CL2*,<br>P_{TEF1}-*VvVST1*),<br>P_{TEF1}-*Sc*ARO7^{G141S}, P_{PGK1}-*ScARO4*^{K229L},<br>P_{TEF1}-*ScACC1*^{S659A,S1157A}<br>Δ*ARO10*, P_{TDH3}-*EcaroL*, P_{TDH3}-*SeACS*^{L641P}	Multi-copy integration of *AtPAL-AtC4H- At4CL1-VvSTS1*	Glucose<br><br>Glucose<br>Ethanol	272.64<br><br>755<br>812	Flask<br>(Batch)<br>Bioreactor<br>(Fed-batch)	[12]
Ethanol Red RBP<br>(Ethanol Red)	*X4*: P_{TDH3}-*AtPAL2*, P_{FBA1}-*AtC4H*, P_{PGK1}-*At4CL2*,<br>P_{TEF1}-*VvVST1*	The first report of lignocellulosic resveratrol production	Glucose +<br>Ethanol<br>Cellulose	187.07<br><br>151.65	Flask<br>(Batch)	[13]

L501<br>(Ethanol Red)	*X4: P_{TDH3}-AtPAL2, P_{FBA1}-AtC4H, P_{PGK1}-At4CL2,*<br>*P_{TEF1}-VvVST1*<br>*P_{PGK1}-KIALC12, P_{TEF1}- KIALC4*	The first report on resveratrol production from lactose, relevant in dairy wastes	Lactose	210	Flask<br>(Batch)	[14]
L543<br>(Ethanol Red)	*XII-4: ScCyb5←PGK1p- TEF1p→AtATR2*<br>*X-4: TDH3p→AtPAL2, FBA1p→AtC4H,*<br>*PGK1p→At4CL2, TEF1p→VvVST1*<br>*X-3: TEF1p→RKI1, TDH3p→RPE1*<br>*XI-3: TEF1p→TKL1, TDH3p→PsTAL1*<br>*XII-2: TEF1p→CpXylA, TDH3p→PsSUT1*<br>*XII-5: TEF1p→CpXylA, TDH3p→PsXYL3*<br>*ΔGRE3::TEF1p→CpXylA*	The first report on the use of renewable carbon sources for resveratrol production from xylose and the use of winery by-products as a substrate to produce this stilbenoid	Glucose + Xylose<br><br>All sugars in wine wastes	388<br><br>161.1 ~ 282.7	Flask<br>(Batch)	[15]
BRT10<br>(*S. cerevisiae*<br>BY4742)	*yorwΔ17*::HIS3/ *T_{ADH1}-VvSTS-P_{PGK1}/ P_{TEF1}-Pc4CL-T_{CYC1}*<br>*ura3*::*T_{ADH1}-RtTAL-P_{PGK1}/P_{TDH3}-VvSTS-T_{CYC1}*/ URA3<br>*cit2*Δ::*T_{ADH1}-RtTAL-P_{PGK1}/P_{TDH3}-VvSTS-T_{CYC1}*<br>*leu2*::*T_{ADH1}- AtCPR1-P_{PGK1}/P_{TDH3}-AtC4H- T_{CYC1}*/LEU2<br>*lpp1*Δ::*T_{ADH1}-RtTAL-P_{PGK1}/P_{TDH3}-VvSTS- T_{CYC1}*<br>*1309a*::*T_{ADH1}-ARO4^{K229L}-P_{PGK1}/P_{TEF1}-ARO7^{G141S}-T_{CYC1}*<br>*511b*::*T_{ADH1}-EcAROL-P_{PGK1}/P_{TEF1}-ARO2-T_{CYC1}*<br>*pdc6*Δ::*P_{TEF1}-ScACC1$^{S659A/S1157A}$-T_{CYC1}*<br>*dpp1*Δ::*T_{ADH1}-VvSTS-P_{PGK1}/P_{TEF1}-Pc4CL-T_{CYC1}*<br>*lys2*::LYS2	Taking the advantage of bi-functional *RtTAL*, enabling the simultaneously use of phenylalanine and tyrosine by only one enzyme;<br>Recover the lysine pathway;<br>A new record for the resveratrol titer in *S. cerevisiae*	Glucose	1155<br><br>4100	Flask<br>(Batch)<br><br>Bioreactor<br>(Fed-batch)	This study

a. Other examples especially start with precursors can refer to some previous reviews [16-18] or reports [9]; *b*. N.A., not available; *c*. strains bearing plasmids for genes expression

Table S2 Plasmids used in this study

**Plasmids**	**Description**	**Reference**
pCas	2μ/pUC, G418/Kan, *S. pyogenese* Cas9	[19]
CIT2-pCas	pCas with *gRNA* specific for site c*it2* gttatggtcatgctgtgcta	This study
LPP1-pCas	pCas with *gRNA* specific for site *lpp1* gccatgacagagatcatcct	This study
PHA2-pCas	pCas with *gRNA* specific for site *pha2* tcagcgacaaaagtaaacag	This study
1309a-pCas	pCas with *gRNA* specific for site *1309a* cctgtggtgactacgtatcc	This study
511b-pCas	pCas with *gRNA* specific for site *511b* cagtgtatgccagtcagcca	This study
PDC6-pCas	pCas with *gRNA* specific for site *pdc6* gatgcgtgcgtaaccatcgg	This study
911b-pCas	pCas with *gRNA* specific for site *911b* gtaatattgtcttgtttccc	This study
DPP1-pCas	pCas with *gRNA* specific for site *dpp1* gatcgttgccaacctgttga	This study
G418	2μ/pUC, G418/Kan, T_{ADH1}- P_{PGK1}/P_{TEF1}-T_{CYC1}	This study
G418-PV	T_{ADH1}-*Pc4CL*-P_{PGK1}/P_{TEF1}-*VvSTS*- T_{CYC1}	This study
G418-TS	T_{ADH1}-*RtTAL*- P_{PGK1}/P_{TDH3}-*VvSTS*- T_{CYC1}	This study
G418-C4H	T_{ADH1}-*AtCPR1*- P_{PGK1}/P_{TDH3}-*AtC4H*- T_{CYC1}	This study
G418-Aro47	T_{ADH1}-*ARO4*^{K229L} - P_{PGK1}/P_{TEF1}-*ARO7*^{G141S} - T_{CYC1}	This study
G418-AroL2	T_{ADH1}-*EcAROL* - P_{PGK1}/P_{TEF1}-*ARO2* - T_{CYC1}	This study
G418-ACC1	T_{ADH1}- P_{PGK1}/P_{TEF1}-*ScACC1*$^{S659A/S1157A}$ - T_{CYC1}	This study

Table S3 Primers used in this study

1.1 Primers used to construct plasmids with expression cassettes

Primers	Sequence (5'-3')	Notes
T_{CYC1}-F	tcatgtaattagttatgtcacgcttaca	For G418 backbone
T_{ADH1}-R	agttataaaaaaaataagtgtatacaaattttaaag	
G418-PV-F1	cacttatttttttataacttttactttggcaaatcaccagagg	
G418-PV-R1	atgggtgattgtgttgctcca	DNA fragments for G418-PV
G418-PV-F2	ggagcaacacaatcacccattgttttatatttgttgtaaaaagtagataattact	
G418-PV-R2	cttcaacggaagccatttgtaattaaaacttagattagattgctatg	
G418-PV-F3	caaaatggcttccgttgaagaattca	
G418-PV-R3	tgacataactaattacatgatcaattggtaacggttggaaca	
G418-TS-F0	atggcttccgttgaagaattca	Backbone
G418-TS-F1	cacttatttttttataactttaagccaacattttcaataaaacatt	
G418-TS-R1	acaatggctcctagaccaactagtcaaa	DNA fragments for G418-TS
G418-TS-F2	gttggtctaggagccattgttttatatttgttgtaaaaagtagataattact	
G418-TS-R2	caacgctagtatacgcacagatattataacatctgcac	
G418-TS-F3	ctgtgcgtatactagcgttgaatgttagcgtca	
G418-TS-R3	aattcttcaacggaagccatttgtttgtttatgtgtgtttattcga	
G418-C4H-F1	cacttatttttttataacttcaccagacatctctgaggtatcttc	
G418-C4H-R1	atgacttctgctttgtatgcttccg	DNA fragments for G418-C4H
G418-C4H-F2	gcatacaaagcagaagtcattgttttatatttgttgtaaaaagtagataattact	
G418-C4H-R2	ccaacaacaacaagtccatttgtttgtttatgtgtgtttattcga	
G418-C4H-F3	aatggacttgttgttgttggaaaag	
G418-C4H-R3	tgacataactaattacatgatcaacagtttcttggcttcataacg	
G418-Aro47-F1	cacttatttttttataactctatttcttgttaacttctcttctttgtctg	DNA fragments for G418-Aro47
G418-Aro47-R1	gggtgttactctacatggtgttgctgctatcacc	
G418-Aro47-F2	caccatgtagagtaacacccatgaaatggtgaga	
G418-Aro47-R2	caatgagtgaatctccaatgttcgc	
G418-Aro47-F3	cattggagattcactcattgttttatatttgttgtaaaaagtagataattact	
G418-Aro47-R3	ctggttttgtgaaatccatttgtaattaaaacttagattagattgctatg	
G418-Aro47-F4	aatggatttcacaaaaccagaaactg	
G418-Aro47-R4	ctctagtggcaacagaactgaagttattcttatcatcaccatctcttt	
G418-Aro47-F5	cagttctgttgccactagagatatagaatg	
G418-Aro47-R5	tgacataactaattacatgattactcttccaaccttcttagcaagt	
G418-AroL2-F1	cacttatttttttataacttcaacaattgatcgtctgtgcc	DNA fragments for G418-AroL2
G418-AroL2-R1	taaaacaatgacacaacctctttttctgatcg	
G418-AroL2-F2	gaggttgtgtcattgttttatatttgttgtaaaaagtagataattact	
G418-AroL2-R2	cccaaacgttgacatttgtaattaaaacttagattagattgctatg	
G418-AroL2-F3	acaaaatgtcaacgtttgggaaactgtt	
G418-AroL2-R3	tgacataactaattacatgattaatgaaccacggatctggaga	
G418-ACC1-R0	tttgtaattaaaacttagattagattgctatg	Backbone

1.1 Primers used to construct plasmids with expression cassettes (continued)

**Primers**	**Sequence (5'-3')**	**Notes**
G418-ACC1-F1	atctaagttttaattacaaaatgagcgaagaaagcttattcga	DNA fragments for G418-ACC1
G418-ACC1-R1	accatcagctagttgacgcagtatgatatcacattta	
G418-ACC1-F2	tgcgtcaactagctgatggtggtcttttgattgc	
G418-ACC1-R2	tgaaacagcaacagccctgttcatacccatt	
G418-ACC1-F3	acagggctgttgctgtttcagatttgtcatatgttgc	
G418-ACC1-R3	tgacataactaattacatgattatttcaaagtcttcaacaatttttctt	

2.1 Primers used to construct plasmids pCas of specific editing sites

**Primers**	**Sequence (5'-3')**	**Notes**
CIT2-pCas-F	gttatggtcatgctgtgctagttttagagctagaaatagc	
CIT2-pCas-R	tagcacagcatgaccataacaaagtcccattcgccaccc	
LPP1-pCas-F	gccatgacagagatcatcctgttttagagctagaaatagc	
LPP1-pCas-R	aggatgatctctgtcatggcaaagtcccattcgccaccc	
PHA2-pCas-F	tcagcgacaaaagtaaacaggttttagagctagaaatagc	
PHA2-pCas-R	ctgtttacttttgtcgctgaaaagtcccattcgccaccc	
1309a-pCas-F	cctgtggtgactacgtatccgttttagagctagaaatagc	
1309a-pCas-R	ggatacgtagtcaccacaggaaagtcccattcgccaccc	
511b-pCas-F	cagtgtatgccagtcagccagttttagagctagaaatagc	
511b-pCas-R	tggctgactggcatacactgaaagtcccattcgccaccc	
PDC6-pCas-F	gatgcgtgcgtaaccatcgggttttagagctagaaatagc	
PDC6-pCas-R	ccgatggttacgcacgcatcaaagtcccattcgccaccc	
911b-pCas-F	gtaatattgtcttgtttcccgttttagagctagaaatagc	
911b-pCas-R	gggaaacaagacaatattacaaagtcccattcgccaccc	
DPP1-pCas-F	gatcgttgccaacctgttgagttttagagctagaaatagc	
DPP1-pCas-R	tcaacaggttggcaacgatcaaagtcccattcgccaccc	

3.1 Primers used to amplify fragments to genomic editing in BY4742

**Primers**	**Sequence (5'-3')**	**Notes**
YORW-Up-F	tgtgcacaaaggccataatattatg	Fragments to form BR1
YORW-Up-R	attaccgaggcataaaaaaatatagagtgtactaggatggcatgagttatggttg	
Y-HIS3-F	ttggtaactgtgcaaccataactcatgccatcctagtacactctatattttttatg	
Y-HIS3-R	ctcttattgaccacacctctaccggcatgccgactacataagaacacctttggtg	
Y-TADH1-F	acgatgttccctccaccaaaggtgttcttatgtagtcggcatgccggtagaggtg	
Y-TCYC1-R	gaattttgagagcccacttttgttggggacgattgcaaattaaagccttcgagc	
YORW-Down-F	ggttttgggacgctcgaaggctttaatttgcaatcgtccccaacaaaagtg	
YORW-Down-R	aaagctggctccccttagac	
△PHA2-UP-F	acatactaccttgcagttcc	Fragments to delete *pha2*
△PHA2-UP-R	accccacaggcaccactgtttacttttgtcgctgataattgaaggatgaatgcgg	
△PHA2-Down-F	aacttcattatatttccgcattcatccttcaattaatgcctgggatttcttgacg	
△PHA2-Down-R	aatcgtgtcgtgtgttcgac	

3.1 Primers used to amplify fragments to genomic editing in BY4742 (Continued)

**Primers**	**Sequence (5'-3')**	**Notes**
Ura3-Up-F	gtgaatttgcagtggtaacg	Fragments
Ura3-Up-R	tcttattgaccacacctctaccggcatgccgacactatctcttagcatctttaatc	to form
Ura3- TADH1-F	gtagaaaaggattaaagatgctaagagatagtgtcggcatgccggtagaggtg	BRT2
Ura3-TCYC1-R	aaaaatgatgaattgaaattaagggttctcgaggcaaattaaagccttcgagcgtc	
CYC-UraM-F	gaaggttttgggacgctcgaaggctttaatttgcctcgagaacccttaatttcaattc	
Leu-UraM-R	ccgagattcccggggcagtggattcaagtgctctgaagctctaatttgtgagtttag	
Ura3-Down-F	cacaaattagagcttcagagcacttgaatccactgccccgggaatctcggtcgtaa	
Ura3-Down-R	tctttatatttacatgctaaaaatgggc	
Leu2-Up-F	acatcgagaccaagaagaacattgc	
Leu2-Up-R	atgccgatacaatccttgcccgtgatgataatatatagtagtaacctgaaaaatag	
Leu- TADH1-F	actactatatattatcatcacgggcaaggattgtatcggcatgccggtagaggtg	Fragments
Leu-TCYC1-R	catcttacgatacctgagtattcccacagttgcaaattaaagccttcgagcgtc	to form
CYC-LEUM-F	gaaggttttgggacgctcgaaggctttaatttgcaactgtgggaatactcaggtatc	BRT3
Leu-LEUM-R	atgttaaagtgcaattctttttccttatcacgtctaccctatgaacatattccattttg	
Leu2-Down-F	aaattacaaaatggaatatgttcatagggtagacgtgataaggaaaaagaattgc	
Leu-Down-R	cataccacgttgaacggatc	
CIT2-Up-F	agttgttgccacaacataag	Fragments
CIT2-Up-R	gctcttattgaccacacctctaccggcatgccgaggaactgtcatttttcttgttac	to integrate
CIT2-tADH-F	ttaataatactagtaacaagaaaaatgacagttcctcggcatgccggtagaggtg	at site *cit2*
CIT2-tCYC1-R	cagaggggtgtaaaagtaggatgtaatccgcaaattaaagccttcgagcgtc	
CIT2-Down-F	gaaggttttgggacgctcgaaggctttaatttgcggattacatcctacttttac	
CIT2-Down-R	gaacgtaaaacaagtaaaaatgtag	
LPP1-Up-F	cttatacgtctcccaatcatg	Fragments
LPP1-Up-R	gctcttattgaccacacctctaccggcatgccgatatgacgagtttccttaggc	to integrate
LPP1-tADH-F	tcctacatcaacgcctaaggaaactcgtcatatcggcatgccggtagaggtg	at site *lpp1*
LPP1-tCYC1-R	gcttttattctttcctgataggactctgtaagtgcaaattaaagccttcgagcgtc	
LPP1-Down-F	gaaggttttgggacgctcgaaggctttaatttgcacttacagagtcctatcagg	
LPP1-Down-R	gaagcaagattctcgaaaatac	
1309a-UP-F	cgttgttgatcttgtagtctg	Fragments
1309a-UP-R	cgctcttattgaccacacctctaccggcatgccgagatcctaaactgcgtcatag	to integrate
1309a-ADH1-F	atcaaagaaacttactatgacgcagtttaggatctcggcatgccggtagaggtg	at site *1309a*
1309a-CYC1-R	ataaacaaggggctttacgatggagtagtagagcaaattaaagccttcgagcgtc	
1309a-Down-F	gaaggttttgggacgctcgaaggctttaatttgctctactactccatcgtaaagcc	
1309a-Down-R	cggcaaaattaaacgaaaacc	
511b-Up-F	gcaacggtttttgaatgctatg	Fragments
511b-UP-R	ctcttattgaccacacctctaccggcatgccgagaaatctgtaccaaccgtatagg	to integrate
511b-ADH1-F	agggtctctttcacctatacggttggtacagatttctcggcatgccggtagaggtg	at site *511b*
511b-CYC1-R	aagaaaatagaagcaaacgacgtaatgccggcaaattaaagccttcgagcgtc	
511b-Down-F	gaaggttttgggacgctcgaaggctttaatttgccggcattacgtcgtttgcttc	
511b-Down-R	caccagcaaagtcaacctcagag	

3.1 Primers used to amplify fragments to genomic editing in BY4742 (Continued)

**Primers**	**Sequence (5'-3')**	**Notes**
911b-UP-F	atggtcgagaagatgagatatg	Fragments to integrate at site *911b*
911b-UP-R	gctcttattgaccacacctctaccggcatgccgacatttatatttatgcccattcaac	
TADH1-F	tcggcatgccggtagaggtg	
PTDH3-R	atactagcgttgaatgttagcg	
911b-Down-F	gttgttgacgctaacattcaacgctagtatcctggagaagtaaatgaaaaatg	
911b-Down-R	ctcaattttcccttttgtcatc	
PDC6-Up-F	aatgttatagagttcacacc	Fragments to integrate at site *pdc6*
PDC6-Up-R	acattttgaagctatggtgtgtgggggatcactttgttggcaatatgtttttgc	
PDC6-TEF1-F	cacgtaatatagcaaaaacatattgccaacaaaagtgatcccccacacaccatag	
PDC6-CYC1-R	caacaataattcgtttgagtacactactaatggcgcaaattaaagccttcgagcgtc	
PDC6-Down-F	agaaggttttgggacgctcgaaggctttaatttgcgccattagtagtgtactcaaac	
PDC6-Down-R	gagcacttgttatttataaaaag	
DPP1-Up-F	atagttttccagtagtgcac	
DPP1-Up-R	gctcttattgaccacacctctaccggcatgccgaggtcgtttgctatgatttaattc	Fragments to integrate at site *dpp1*
DPP1-tADH-F	gcaaagaatcagaattaaatcatagcaaacgacctcggcatgccggtagaggtg	
DPP1-tCYC1-R	aatacgtatatttcgtatgtcatgtggagtatatagcaaattaaagccttcgagcgtc	
DPP1-Down-F	gaaggttttgggacgctcgaaggctttaatttgctatatactccacatgacatacg	
DPP1-Down-R	acatagtatgtgttaagggg	
Lys2-F	acaaaggaaagcagttgctttc	Complement of Lys2
Lys2-R	gtggagattgaaaagagctg	

Table S4 Cell densities and resveratrol titers of strains BRT9 and BRT10 cultured in YPD medium

**Strains**	**YPD-20G**		**YPD-40G**	
	**OD_{600}**	**Resveratrol (mg/L)**	**OD_{600}**	**Resveratrol (mg/L)**
BRT9	15.75±0.38*	486.68±7.25	24.48±0.03	1139.94±63.34
BRT10	16.43±0.17	505.75±12.31	25.71±0.50	1078.81±53.97

Note: YPD-20G, YPD medium with 20 g/L glucose; YPD-40G, YPD medium with 40 g/L glucose. Statistical analysis was performed by using Student's t-test (two tailed; two-sample assuming equal variance; *p<0.05, and signs was not shown when p>0.05), the result of strain BRT9 was compared with strain BRT8, and the result of bRT10 was compared with strain BRT9. The displayed average values and standard deviations were calculated from three independent biological experiments.

Supplementary Figures

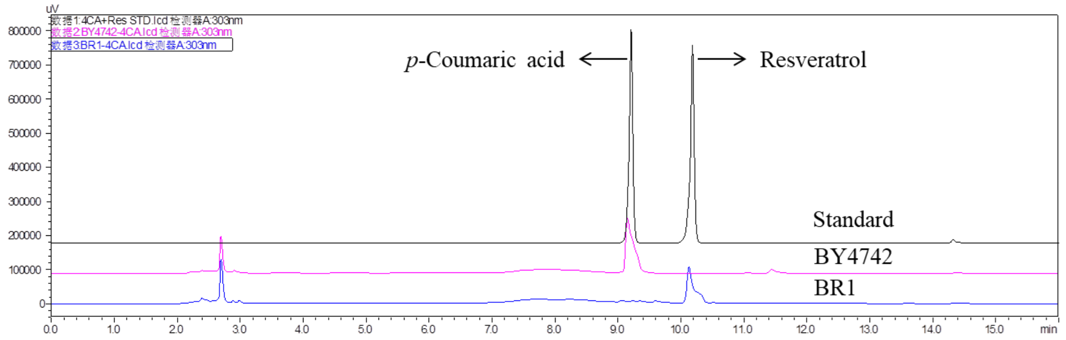

Fig. S1 Production of resveratrol in engineered strain BR1. Standards of *p*-coumaric acid and resveratrol, and samples of strain BR4742 and BR1 were analyzed by HPLC. Standards were dissolved in methanol, and fermentative broths of strain BY4742 and BR1 were mixed with ethanol. Strains were cultured in YPD medium with 0.5 mmol/L *p*-coumaric acid for 72 h.

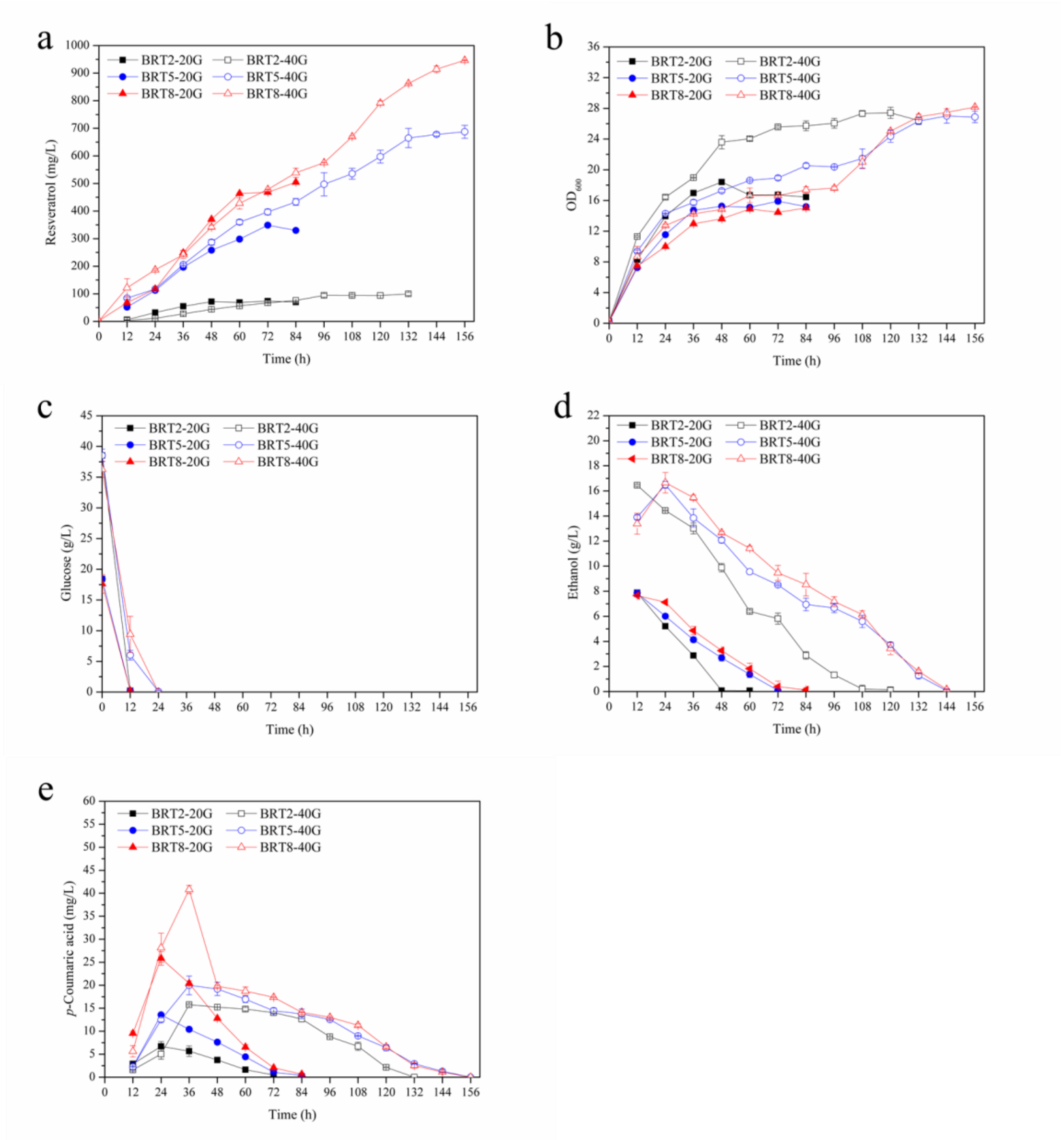

Fig. S2 Time courses of (a) resveratrol production, (b) cell growth, (c) glucose consumption, (d) ethanol concentration and (e) *p*-coumaric acid concentration of strains BRT2, BRT5 and BRT8 cultured in YPD medium with 20 g/L and 40 g/L glucose respectively. Error bars represent standard deviations from three independent biological experiments.

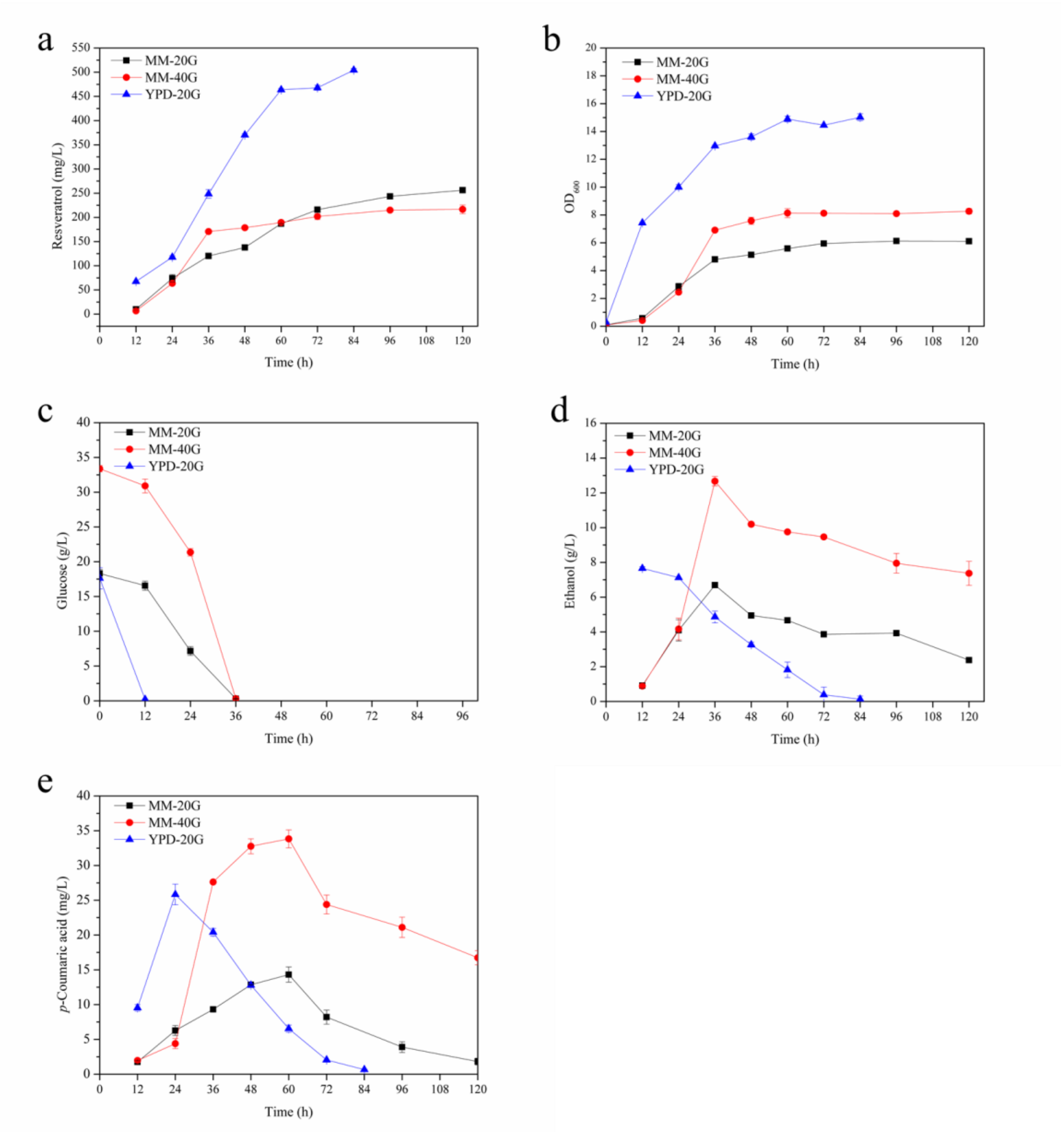

Fig. S3 Time courses of (a) resveratrol production, (b) cell growth, (c) glucose consumption, (d) ethanol concentration and (e) *p*-coumaric acid concentration of strain BRT8 cultured in different media. Error bars represent standard deviations from three independent biological experiments.

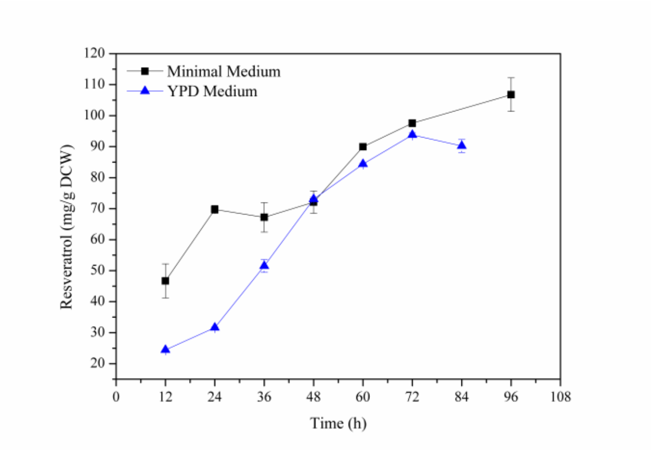

Fig. S4 Time course of resveratrol yield on dry cell weight when strain BRT8 was cultured in YPD or minimal medium. DCW: dry cell weight. Error bars represent standard deviations from three independent biological experiments.

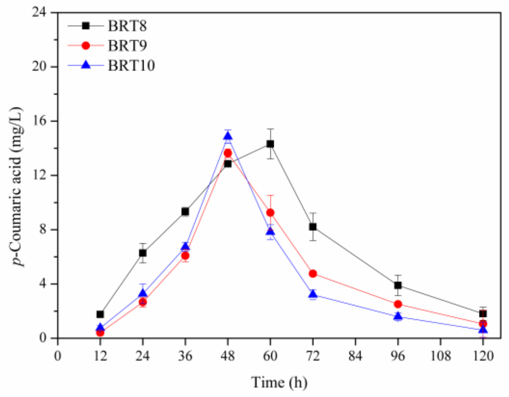

Fig. S5 Time course of *p*-coumaric acid concentration of strains BRT8, BRT9 and BRT10 cultured in minimal medium. 0.5 g/L lysine was added in the minimal medium for the growth of strain BRT8 and BRT9. Error bars represent standard deviations from three independent biological experiments.

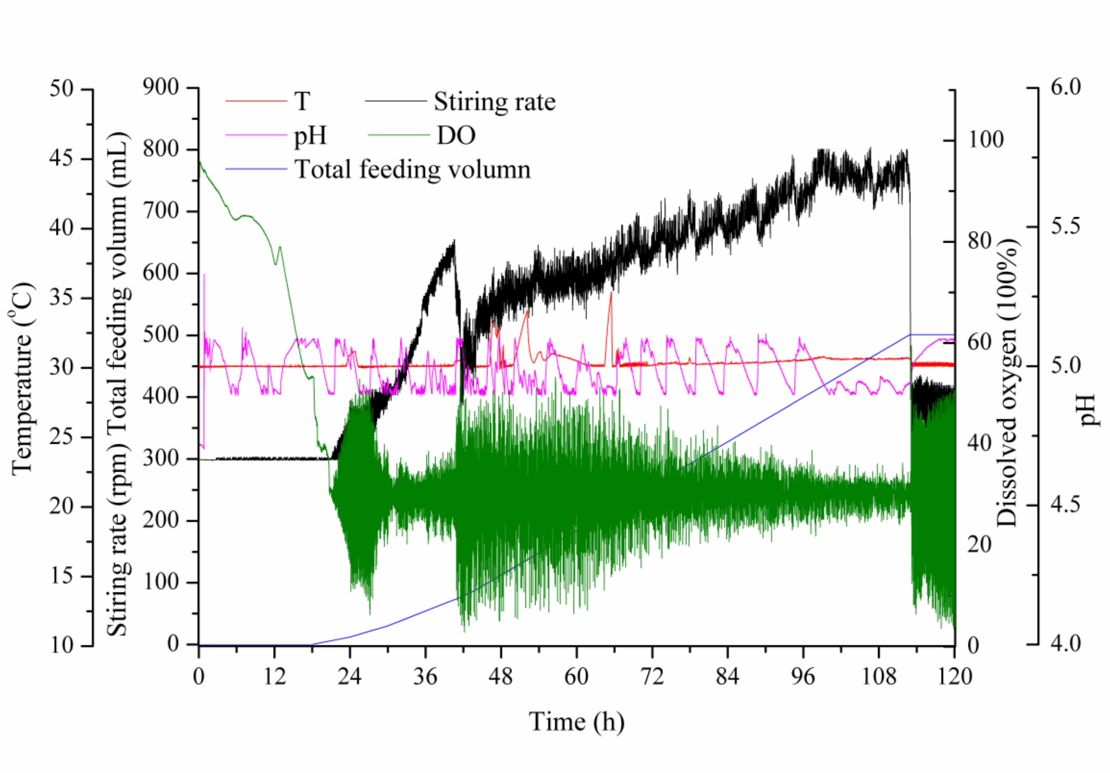

Fig. S6 Parameters during the fed-batch fermentation in the bioreactor using the non-auxotrophic strain BRT10 in minimal medium.

References

1. Shrestha A, Pandey RP, Pokhrel AR, Dhakal D, Chu LL, Sohng JK. Modular pathway engineering for resveratrol and piceatannol production in engineered *Escherichia coli*. Appl Microbiol Biotechnol. 2018;102(22):9691-706.
2. Wu J, Zhou P, Zhang X, Dong M. Efficient de novo synthesis of resveratrol by metabolically engineered *Escherichia coli*. J Ind Microbiol Biotechnol. 2017;44(7):1083-95.
3. Cotner M, Zhan J, Zhang Z. A computational metabolic model for engineered production of resveratrol in *Escherichia coli*. ACS Synth Biol. 2021;10(8):1992-2001.
4. Liu X, Lin J, Hu H, Zhou B, Zhu B. *De novo* biosynthesis of resveratrol by site-specific integration of heterologous genes in *Escherichia coli*. FEMS Microbiol Lett. 2016;363(8).
5. Kumokita R, Bamba T, Inokuma K, Yoshida T, Ito Y, Kondo A, et al. Construction of an L-tyrosine chassis in *Pichia pastoris* enhances aromatic secondary metabolite production from glycerol. ACS Synth Biol. 2022;11(6):2098-107.
6. Kobayashi Y, Inokuma K, Matsuda M, Kondo A, Hasunuma T. Resveratrol production from several types of saccharide sources by a recombinant *Scheffersomyces stipitis* strain. Metab Eng Commun. 2021;13:e00188.
7. He Q, Szczepanska P, Yuzbashev T, Lazar Z, Ledesma-Amaro R. De novo production of resveratrol from glycerol by engineering different metabolic pathways in *Yarrowia lipolytica*. Metab Eng Commun. 2020;11:e00146.
8. Gu Y, Ma J, Zhu Y, Ding X, Xu P. Engineering *Yarrowia lipolytica* as a chassis

for *de novo* synthesis of five aromatic-derived natural products and chemicals. ACS Synth Biol. 2020;9(8):2096-106.

9. Saez-Saez J, Wang G, Marella ER, Sudarsan S, Cernuda Pastor M, Borodina I. Engineering the oleaginous yeast *Yarrowia lipolytica* for high-level resveratrol production. Metab Eng. 2020;62:51-61.

10. Liu M, Wang C, Ren X, Gao S, Yu S, Zhou J. Remodelling metabolism for high-level resveratrol production in *Yarrowia lipolytica*. Bioresour Technol. 2022;365:128178.

11. Li M, Kildegaard KR, Chen Y, Rodriguez A, Borodina I, Nielsen J. De novo production of resveratrol from glucose or ethanol by engineered *Saccharomyces cerevisiae*. Metab Eng. 2015;32:1-11.

12. Li M, Schneider K, Kristensen M, Borodina I, Nielsen J. Engineering yeast for high-level production of stilbenoid antioxidants. Sci Rep. 2016;6:36827.

13. Costa CE, Moller-Hansen I, Romani A, Teixeira JA, Borodina I, Domingues L. Resveratrol production from hydrothermally pretreated eucalyptus wood using recombinant industrial *Saccharomyces cerevisiae* strains. ACS Synth Biol. 2021;10(8):1895-903.

14. Costa CE, Romani A, Teixeira JA, Domingues L. Resveratrol production for the valorisation of lactose-rich wastes by engineered industrial *Saccharomyces cerevisiae*. Bioresour Technol. 2022;359:127463.

15. Costa CE, Romaní A, Møller-Hansen I, Teixeira JA, Borodina I, Domingues L. Valorisation of wine wastes by *de novo* biosynthesis of resveratrol using a

recombinant xylose-consuming industrial *Saccharomyces cerevisiae* strain. Green Chem. 2022.

16. Feng C, Chen J, Ye W, Liao K, Wang Z, Song X, et al. Synthetic biology-driven microbial production of resveratrol: Advances and perspectives. Front Bioeng Biotechnol. 2022;10:833920.

17. Ibrahim GG, Yan J, Xu L, Yang M, Yan Y. Resveratrol production in yeast hosts: Current status and perspectives. Biomolecules. 2021;11(6).

18. Shrestha A, Pandey RP, Sohng JK. Biosynthesis of resveratrol and piceatannol in engineered microbial strains: achievements and perspectives. Appl Microbiol Biotechnol. 2019;103(7):2959-72.

19. Reider Apel A, d'Espaux L, Wehrs M, Sachs D, Li RA, Tong GJ, et al. A Cas9-based toolkit to program gene expression in *Saccharomyces cerevisiae*. Nucleic Acids Res. 2017;45(1):496-508.
